# Condensins Exert Force on Chromatin-Nuclear Envelope Tethers to Mediate Nucleoplasmic Reticulum Formation in *Drosophila melanogaster*

**DOI:** 10.1534/g3.114.015685

**Published:** 2014-12-30

**Authors:** Julianna Bozler, Huy Q. Nguyen, Gregory C. Rogers, Giovanni Bosco

**Affiliations:** * Geisel School of Medicine at Dartmouth, Hanover, New Hampshire 03755; † Department of Cellular and Molecular Medicine, University of Arizona Cancer Center, University of Arizona, Tucson, Arizona 85724

**Keywords:** nuclear architecture, chromatin force, nucleus, chromatin compaction, nuclear envelope

## Abstract

Although the nuclear envelope is known primarily for its role as a boundary between the nucleus and cytoplasm in eukaryotes, it plays a vital and dynamic role in many cellular processes. Studies of nuclear structure have revealed tissue-specific changes in nuclear envelope architecture, suggesting that its three-dimensional structure contributes to its functionality. Despite the importance of the nuclear envelope, the factors that regulate and maintain nuclear envelope shape remain largely unexplored. The nuclear envelope makes extensive and dynamic interactions with the underlying chromatin. Given this inexorable link between chromatin and the nuclear envelope, it is possible that local and global chromatin organization reciprocally impact nuclear envelope form and function. In this study, we use *Drosophila* salivary glands to show that the three-dimensional structure of the nuclear envelope can be altered with condensin II-mediated chromatin condensation. Both naturally occurring and engineered chromatin-envelope interactions are sufficient to allow chromatin compaction forces to drive distortions of the nuclear envelope. Weakening of the nuclear lamina further enhanced envelope remodeling, suggesting that envelope structure is capable of counterbalancing chromatin compaction forces. Our experiments reveal that the nucleoplasmic reticulum is born of the nuclear envelope and remains dynamic in that they can be reabsorbed into the nuclear envelope. We propose a model where inner nuclear envelope-chromatin tethers allow interphase chromosome movements to change nuclear envelope morphology. Therefore, interphase chromatin compaction may be a normal mechanism that reorganizes nuclear architecture, while under pathological conditions, such as laminopathies, compaction forces may contribute to defects in nuclear morphology.

During the past several decades we have begun to appreciate the structural and functional complexity of the nuclear envelope. Although primarily known for its role in the partitioning of genomic and transcriptional components away from the cytoplasm, the nuclear envelope plays a vital and dynamic role in gene expression, signal transduction, DNA damage repair, and response to mechanosensory inputs (De Vosse *et al.* 2011; Maniotis *et al.* 1997; Therizols *et al.* 2006). The well-described physical structures of the double membrane layer and nuclear structural filaments, such as nuclear lamins, remain the predominant platforms for interaction between chromatin and the cytoplasm (Shimi *et al.* 2012). These two membrane layers are connected through integral membrane proteins and are punctuated with the nuclear pore complex that facilitates bidirectional macromolecule movement between nucleoplasm and cytoplasm. The internal structural support of the nucleus is provided in large part by the nuclear lamina, a meshwork of lamin A- and B-type polymers. Studies of nuclear structure have exposed tissue- specific changes in the threee-dimentional architecture of the nuclear envelope, revealing intricate patterns of tunneling and branching of the nuclear envelope within the nuclear space (Dupuy-Coin *et al.* 1986; Fricker *et al.* 1997). This higher order arrangement of the nuclear envelope has been given the name nucleoplasmic reticulum (NR) (Malhas *et al.* 2011). The importance of these NR structures for chromosome partitioning, nuclear transport, gene expression, or other cellular processes are not well understood. Although we lack a comprehensive understanding of the functional role of nuclear architecture, it has become clear that the nuclear envelope and its three-dimensional shape can profoundly impact genome organization and gene expression (De Vosse *et al.* 2011; Schneider and Grosschedl 2007).

The typical interphase genome is radially organized with heterochromatin and transcriptionally silent gene regions predominantly restricted to the nuclear periphery (Hochstrasser and Sedat 1987; Rajapakse and Groudine 2011). However, exceptions to this trend exist. For example, nuclear pore complexes can recruit and tether transcriptionally active genes (Capelson *et al.* 2010; Rodrıguez-Navarro *et al.* 2004). These observations have led to a model in which association with the nuclear envelope serves as a boundary between active and inactive chromatin (Bermejo *et al.* 2011; Blobel 1985). Other models propose that chromatin tethers to the nuclear envelope serve to organize chromosomes into distinct territories (Bauer *et al.* 2012; Verdaasdonk *et al.* 2013). Regardless of the functional role chromatin-envelope interactions may have, it is clear that chromatin is tethered to the inner nuclear envelope through a variety of DNA-protein and protein-protein interactions (Dahl *et al.* 2005; Gruenbaum *et al.* 2005; Mazumder *et al.* 2008). Chromatin interacts with the nuclear envelope through contacts with the nuclear lamins or the nuclear pore complex (Ye *et al.* 1997). Lamins bind chromatin through interactions with histones or by directly binding sequence-specific regions of DNA termed matrix-attachment regions (Zhao *et al.* 1996). These chromatin-envelope tethers facilitate gene regulation as well as genome organization (Dialynas *et al.* 2010; Therizols *et al.* 2006; Verdaasdonk *et al.* 2013).

Given the substantial role of the nuclear envelope in genome regulation, it is not surprising that defects in nuclear architecture are linked to a variety of diseases. There are 12 known disorders that arise from mutations in the A-type lamins and lamin-associated proteins, such as Emery-Dreifuss muscular dystrophy and Hutchinson-Gilford progeria syndrome (Goldman *et al.* 2004; Sullivan *et al.* 1999). In these syndromes, the characteristic deformation of the nuclear envelope results in a loss of peripheral heterochromatin, altered gene expression (Goldman *et al.* 2004), and disorganization of the genome (Goldman *et al.* 2004; Shumaker *et al.* 2006). Interestingly, the accumulation of aberrantly spliced lamins can occur over time in normal tissues, linking similar changes in nuclear structure with the process of ageing (Scaffidi and Misteli 2006). Additionally, numerous cancers are characterized through their envelope morphology, although in such instances the direct causes of nuclear defects are less clear (Chow *et al.* 2012).

Two major models have surfaced to address the cause of nuclear deformations. The first model proposes that the mechanical properties of the lamins themselves, coupled with their asymmetric distribution, are responsible for nuclear ”blebbing” and invagination of the envelope (Funkhouser *et al.* 2013). A second model suggests that the cytoskeleton stabilizes nuclear architecture by counterbalancing internal forces of the nuclear lamina and chromatin, and only when these stabilizing factors are altered does the nuclear architecture change (Dahl *et al.* 2005; Lammerding *et al.* 2006; Maniotis *et al.* 1997; Mazumder *et al.* 2008). This model allows for dynamic counterbalancing forces to maintain normal architecture, but which could be regulated to form complex nuclear structures in nondisease tissues (Mammoto and Ingber 2010; Mazumder *et al.* 2010). Evidence exists for each model (Rowat *et al.* 2008); however, a comprehensive model of nuclear architecture will likely be complex and include several unidentified factors.

Cytoskeletal forces have been studied in the context of nuclear architecture (Maniotis *et al.* 1997), but the role of chromatin has only begun to be implicated in the mechanical stabilization of nuclear architecture. Recent studies have revealed that decondensation of chromatin directly leads to a mechanically destabilized nucleus (Mazumder *et al.* 2008). Additionally, it has been shown that chromatin acts as a force-bearing element in the unchallenged nucleus (Dahl *et al.* 2005). This has led to the supposition that nuclear and cytoplasmic anchors are important contributors to the structural integrity of the nucleus. Further, it has been suggested that chromatin-envelope interactions constitute a mechanical scaffold that defines and preserves nuclear architecture (Mazumder and Shivashankar 2007; Mazumder *et al.* 2008). Mounting evidence suggests that mechanical forces within the nucleus may have an equally profound effect on nuclear structure and yet remain relatively enigmatic (Buster *et al.* 2013; George *et al.* 2014).

Importantly, dynamic forces exist within the nucleus, not only in preparation for cell division, but during interphase as well. The condensin complexes are recognized as regulators of chromatin structure and topology during cell division, with condensin II maintaining activity during interphase. Shared between condensin I and condensin II is the SMC2/4 heterodimer, endowing the complex with its essential ATPase domains (Kimura and Hirano 1997). Unique to condensin II are the chromosome-associating protein subunits Cap-D3, Cap-H2, and Cap-G2, the regulation of which likely confers interphase activity (Buster *et al.* 2013; Hartl *et al.* 2008; Hirano *et al.* 1997). During interphase, condensin II participates in the maintenance of chromosome territories and drives homolog unpairing (Bauer *et al.* 2012; Hartl *et al.* 2008). Hyperactivation of condensin II, either by inactivation of the negative-regulator SCF^Slimb^ or by overexpression of Cap-H2, results in dramatic chromosome condensation, suggesting that dynamic chromatin movements occur during interphase and that these movements are induced by condensin II activity (Bauer *et al.* 2012; Hartl *et al.* 2008). Similarly, loss of condensin II function leads to lengthening of interphase chromosomes (Bauer *et al.* 2012). Therefore, condensin II could be seen as more broadly regulating the mechanical properties of chromatin mediated support of the nuclear envelope. Notably, in *Drosophila*, interphase condensin II hyperactivity induces nuclear envelope deformations (Buster *et al.* 2013), and in human cancer cell lines, RNA interference (RNAi) depletion of condensin II results in increased nuclear size and distortions of nuclear shape (George *et al.* 2014). Hence, it has been proposed that condensin II-mediated mechanical force within the interphase nucleus is capable of pulling nuclear membrane structures into the nucleus and contribute to remodeling of nuclear architecture (Wallace and Bosco 2013).

In this study, we use polytene nuclei from *Drosophila melanogaster* salivary glands to investigate whether condensin mediated chromatin compaction could lead to remodeling of the nuclear envelope and induce NR-like structures. We chose salivary gland cells because in third instar larvae these cells are postmitotic and never re-enter the cell cycle. Therefore, this eliminates the possibility that envelope reassembly upon exit from mitosis could contribute to alterations in nuclear envelope structure. Additionally, these nuclei are relatively large, making it possible to visualize chromatin-envelope interactions with standard light microscopy. We observe measurable differences in the NR after chromatin compaction mediated by condensin II. For the first time, the dynamic formation of the NR is observed in live cells, and we find that artificial chromatin tethers are sufficient to pull nuclear envelope structures into the interior of the nucleus. An investigation into the formation of the NR will translate into an understanding of the dynamic nature of nuclear envelope structure and reveal forces that regulate nuclear envelope shape and function.

## Materials and Methods

### *Drosophila* strains

All *Drosophila melanogaster* stocks were maintained at 25° on standard corn meal molasses media, with a 12-hr light/dark cycle. As in previous studies, increased condensin II activity was induced by overexpressing a single subunit, Cap-H2 (Bauer *et al.* 2012; Hartl *et al.* 2008). Two different methods of Cap-H2 overexpression are described here, both of which use the GAL4/UAS system. A heat shock−inducible system was used to allow for precise temporal control of Cap-H2 overexpression. In this fly line, the Hsp70-GAL4 is able to drive expression of endogenous Cap-H2 due to a UAS insertion upstream of the Cap-H2 gene (Bauer *et al.* 2012; Hartl *et al.* 2008). This Cap-H2 overexpression line, referred to as the HS > GAL4 UAS > Cap-H2 line (w*;HS83-GFP-LacI, LacO (60F);Hsp70-GAL4, EY09979), is the primary method of Cap-H2 overexpression used in this study. The corresponding control line, lacking the EY09979 (w*;HS83-GFP-LacI, LacO (60F);Hsp70-GAL4), is denoted as the HS > GAL4 line.

To confirm that the observations made are not dependent on heat shock, and to lend further evidence that the phenotype is not specific to the particular overexpression transgene, the salivary gland driver 43B > GAL4 was used to express Cap-H2 in a tissue-specific manor. This driver line was crossed to transgenic flies containing a UAS construct expressing a Cap-H2-GFP protein (*UAS-Cap-H2-eGFP.1*) (Buster *et al.* 2013). A detailed list of all fly stocks used can be found in Supporting Information, Table S1.

### Immunofluorescence and microscopy

All samples prepared for immunofluorescence were fixed in 4% methanol-free formaldehyde in phosphate-buffered saline (PBS; 0.001% triton X) for 5min. Samples were washed in PBS (0.1% triton X), and blocked with 2% normal goat serum for 2 hr. Primary antibodies were incubated overnight at 4° in 2% normal goat serum. For visualization of the nuclear envelope, mouse anti-Dm0 Lamin was used (Adl 84.12 c; Hybridoma Bank) at a concentration of 1:200, and mouse antibody to the nuclear pore complex was used (mab414; Abcam) at a concentration of 1:2500. Secondary antibodies were either fluorescein isothiocyanate or Cy3 conjugated (Jackson ImmunoResearch), used at a concentration of 1:100 and 1:150, respectively, and were incubated for 2 hr at room temperature. All salivary gland and cell culture experiments were carried out in this fashion unless otherwise specified. For experiments which depended on heat shock induction, vials of late third instar larvae experienced heat shock at 37° for 2 hr, followed by a 2-hr recovery at room temperature; experiments that deviate from this protocol carry specific notation.

The membrane marker wheat germ agglutinin (WGA) was used as an additional membrane stain for select samples, following antibody preparation. Alexa Fluor 647 and Alexa Flour 488 conjugated WGA (Life Technologies) was incubated with a sample for 30 min at room temperature at a concentration of 10 μg/mL. All samples were counter stained with DAPI (36 μM), mounted in VECTASHIELD, and stored at −20° until imaged.

Muscle nuclei of HS > GAL4 UAS > Cap-H2 and HS > GAL4 lines were compared after a 2-hr heat shock and 6-hr recovery. Experiments using the larval muscle−specific driver C57 GAL4 without heat shock induction also were conducted. This driver line was crossed to transgenic flies containing a UAS construct expressing a stabilized Cap-H2-GFP protein, in which the last 23 amino acids of the Cap-H2 protein have been truncated (*UAS-Cap-H2ΔC23-eGFP.3*). This deletes a degradation signal recognized by the Slimb E3-ubiquitin ligase (Buster *et al.* 2013). Body wall dissections were carried out using previously described techniques (Budnik *et al.* 1990) and stained with anti-Dm0 Lamin.

A Nikon A1R SI Confocal microscope was used for all light microscopy. Image averaging of 4x during image capture was used for all images unless otherwise specified. The Nikon software Nikon Elements (V 2.0) was used for three-dimentional projections and for three-dimentional analysis of the nuclear envelope.

### Quantification and characterization of NR in salivary glands

To quantify the observed NR phenotype, immunofluorescence was performed on Cap-H2 overexpressing larvae and compared to control. Vials of third instar larvae of the Cap-H2 overexpression line HS > GAL4 UAS > Cap-H2, and the control line HS > GAL4, underwent heat shock for 1.5 hr at 37°. Salivary glands were dissected in PBS after a 1.5-hour recovery at room temperature. Primary antibody to Lamin as described previously was used for visualization of the nuclear envelope and NR. Five glands were imaged for each of three replicate. Z-stacks were taken for 10 nuclei from each salivary gland, with step size of 0.5 microns. An NR event was tallied when a distinct three-dimentional invagination of the envelope into the nuclear space was identified, with a minimum size cut off of 1 μm; superficial wrinkles and folds of the nuclear envelope were not considered. The number of NR events per nucleus for each gland was determined, and a Welch’s t-test was conducted on the means of the two groups to assess statistical significance. Five additional glands from non-heat shock larvae of each line were imaged in parallel for one replicate. The diameter of NR was classified as the greatest two-dimensional distance of the NR cross-section. Measurements were taken by Nikon Elements for every NR event observed in the HS > GAL4 and HS > GAL4 UAS > Cap-H2 lines. Welch’s T-test was performed to test significance.

A comparison of NR quantification techniques was performed to assess the reliability of NR detection for both the anti-Nup and anti-Lamin antibodies. Salivary glands were collected from larvae of the same heat shock treatment and stained with either anti-Nup or anti-Lamin. For each group, three glands were imaged, totaling ten nuclei per gland.

To visualize nuclear-localizing GFP in relation to NR, flies with the UAS > GFP.nls construct were crossed to the HS > GAL4 UAS > Cap-H2 and HS > GAL4 lines. Third instar larvae were dissected and stained following the described method after a 2-hr heat shock and 6-hr recovery period.

### Detection and quantification of NR in cell culture

Kc cells were maintained in Sf900II media (Life Technologies) at a constant temperature of 25°. To achieve increased condensin II activity in cell culture, Slimb, the negative regulator of Cap-H2 was depleted with RNAi (Buster *et al.* 2013). Control (sk) and gene-specific (Slimb) double-stranded RNA (dsRNA) was generated using the following previously described forward and reverse primer pairs (Buster *et al.* 2013); sk (5′CGCTTTTCTGGATTCATCGAC, 5′TGAGTAACCTGAGGCTATGG), Slimb (5′GGCCGCCACATGCTGCG, 5′CGGTCTTGTTCTCATTGGG). Kc cells, at 50–90% confluency, were treated in parallel with either control or gene-specific dsRNA. Every other day, cells were treated with 10 μg of dsRNA in 1 mL of culture media. The experiment was terminated on the seventh day of treatment, and cells were collected for staining. Treated cells were allowed to adhere to a concanavlin A−coated glass cover slip for 15 min before the media was removed and cover slip was washed three times with PBS. Cells were then fixed in 4% methanol-free formaldehyde and stained for the nuclear pore complex (anti-Nup) and WGA and counterstained with DAPI. An NR event was defined as any distinct structure within the nucleus that was positive for both WGA and anti-Nup. Cells from three RNAi experiments were stained and imaged for each treatment condition, with a minimum of 75 cells counted for each replicate. Z-stacks were compiled from confocal images with a step size of 0.25 microns. The average number of NR events per nucleus was calculated for each replicate. A Welch’s *t*-test was performed to assess significance.

### Transmission electron microscopy

To capture a detailed image of the membrane structure of the NR, transmission electron microscopy was performed on the Cap-H2 overexpression line HS > GAL4 UAS > Cap-H2 and control line HS > GAL4. Vials of third instar larvae from each line underwent heat shock for 1.5 hr at 37°. Salivary glands were dissected in PBS after a 2-hr recovery period at room temperature. Salivary glands were fixed overnight in a solution of 2% glutaraldehyde/2% paraformaldehyde. Samples were then prepared for sectioning following a detailed protocol optimized to preserve the ultrastructure of the tissue, which can be found in the supplemental material (File S4). Thin sections were taken and stained with uranyl acetate aqueous solution and lead citrate. Imaging was performed on a FEI Tecnai F20ST field emission gun transmission electron microscope.

### Chromatin-envelope tether

To determine whether tethering of chromatin to the nuclear envelope impacts NR formation, we introduced a LacI-Lamin C fusion protein into our system (a gift from Laurie Wallrath). This fusion protein is able to relocate a LacO array to the nuclear periphery by binding to the LacO sequence and integrating its C terminus into the nuclear lamina (Dialynas *et al.* 2010). Fly stocks with this tether were crossed to the heat shock-inducible Cap-H2 overexpression line LacO, GFP-LacI; HS > GAL4 UAS > Cap-H2, and the control line LacO, GFP-LacI; HS > GAL4. To account for the frequency of chance associations between the NR and LacO array, an additional cross between LacO, GFP-LacI; HS > GAL4 UAS > Cap-H2 and a control line was tested.

Six salivary glands were dissected for each replicate. Nuclear envelope was visualized by immunofluorescence (IF) using nuclear pore antibody, LacO arrays were visualized by green fluorescent protein (GFP) fluorescence of GFP-LacI introduced from the HS > GAL4 UAS > Cap-H2 and HS > GAL4 line. A minimum of 12 nuclei from each gland were imaged, with z-stacks taken at 0.5-μm steps. An association between the LacO array and NR was considered to be present if the GFP signal was observed at any point along the periphery of the NR event. The proportion of nuclei positive for NR events associating with the LacO array was calculated for each genotype, and χ^2^ analysis conducted to assess statistical significance.

### Weakening of the nuclear envelope

Structural integrity of the nuclear envelope was compromised through expression of an aberrant lamin protein. Expression of aberrant lamin protein was achieved with the use of a UAS-progerin line; a transgenic fly line that expresses the human progerin protein under UAS control (gift from David Tree) (Beard *et al.* 2008). These flies were crossed to the HS > GAL4 UAS > Cap-H2 and HS > GAL4 lines; salivary glands from the progeny were dissected. Salivary glands were stained with antibody to Lamin for identification of the nuclear envelope and NR. Five nuclei were imaged per salivary gland; 15 glands were imaged for all experiments, with five glands for each of the three replicates. Quantification and statistical techniques were otherwise identical to the preceding protocol. Raw data is available in supplemental information, File S5.

### Live cell imaging

Live cell imaging was conducted on salivary gland nuclei. To view the nuclear envelope without fixation, homozygous stocks were made with a GFP-tagged nuclear pore protein, GFP-Nup107 (Bloomington Stock Center stock number 35514), and the EY09979 and heat shock promoter elements from the HS > GAL4 UAS > Cap-H2 line. Late third instar larvae experienced heat shock at 37° for 30 min in standard *Drosophila* media vials. Vials were then removed from the incubator and kept at room temperature for an additional 30 min. Salivary glands were dissected in PBS and placed on a poly-L-lysine−treated cover slip affixed to the bottom of a stainless-steel slide with a 10-mm hole. The glands were submerged in PBS and immediately imaged. Imaging sessions lasted for 45 min; z-stacks were taken with 2-μm step size every 3 min for the duration of the imaging session. Image averaging of 2× was used during image capture for all live cell time lapses. Control imaging was conducted in identical fashion but excluded heat shock.

## Results

### Condensin-mediated forces remodel the nuclear envelope

**Figure 1:**
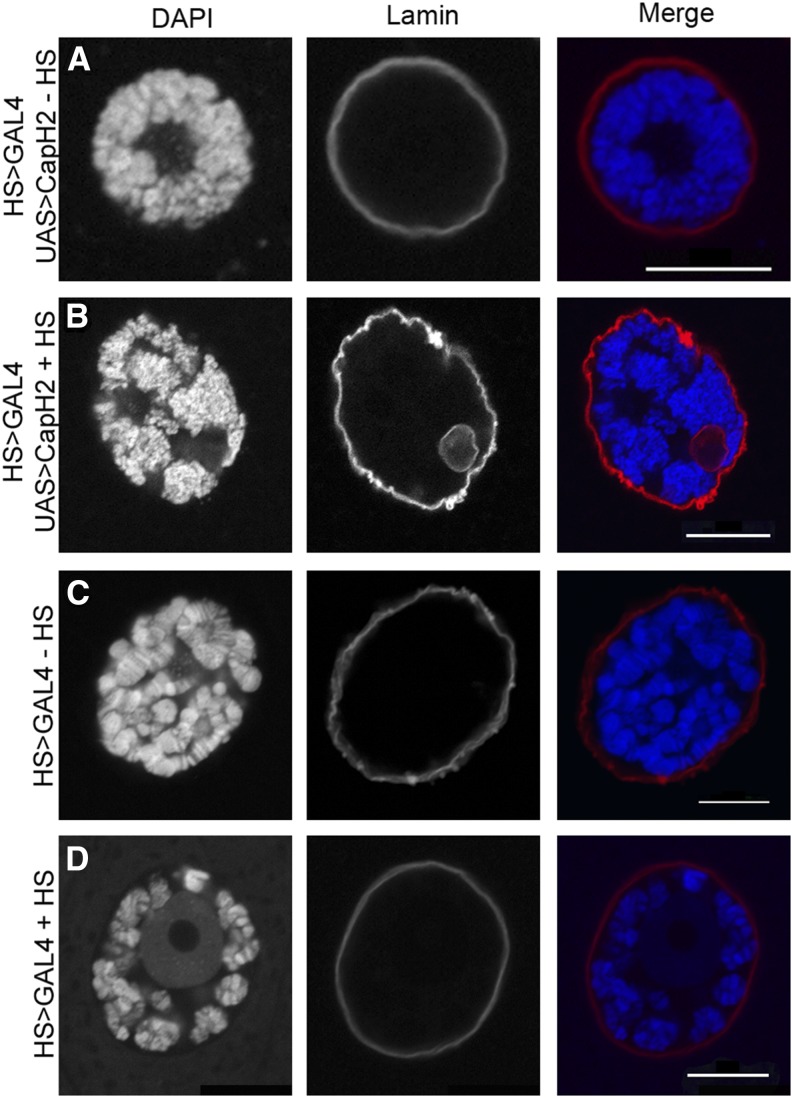
Changes in nuclear architecture are induced by Cap-H2 overexpression. Individual salivary gland nuclei with different heat shock treatments are visualized with DAPI and anti-lamin marking the chromatin and nuclear envelope, respectively. Normal nuclear morphology can be seen in the heat shock inducible Cap-H2 overexpression line in the absence of heat shock treatment (A). Nuclear deformations can be observed in the heat shock−inducible Cap-H2 overexpression line with heath shock treatment (B), where the nuclear lamina has formed structures within the nuclear space. Normal morphology is observed in the HS > GAL4 control line without (C) and with (D) heat shock treatment. In the particular z-slice shown for the heat shock control (D), the nucleolus is especially prominent in the center of the nucleus but is not indicative of a global reorganization of the chromatin to the nuclear periphery. Scale bar is 10 microns for all panels.

**Figure 2:**
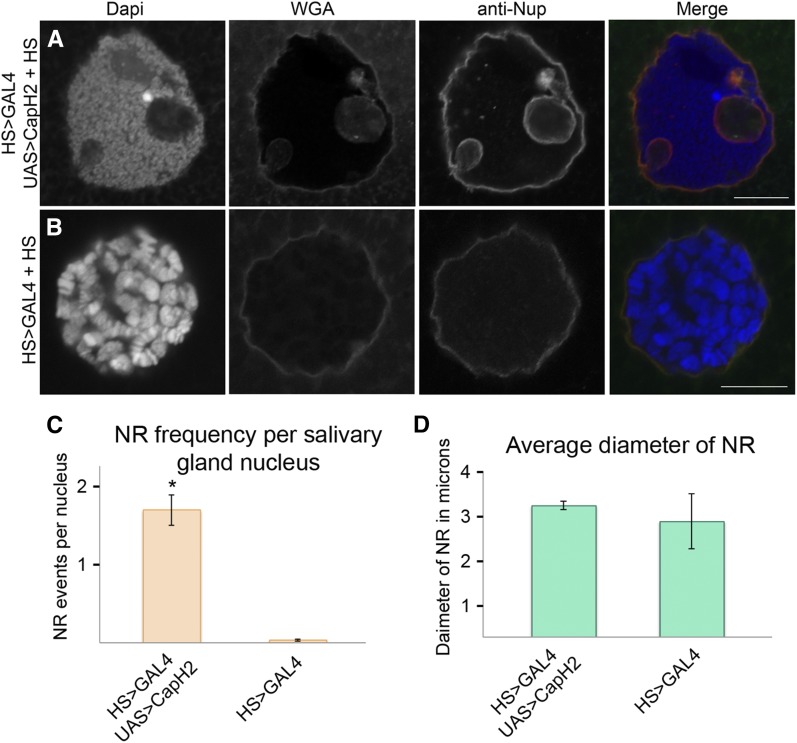
Cap-H2 overexpression leads to remodeling of the nuclear membrane. The nuclear membrane of individual salivary gland nuclei is visualized with wheat germ agglutinin (WGA) and antibody to the nuclear pore complex (anti-Nup). Formation of nuclear membrane structures within the nucleus can be seen in Cap-H2 overexpression, induced by heat shock (A). Typical spherical nuclear membrane structure, with absence of intranuclear structures, can be seen in GAL4 control with heat shock treatment (B). Quantification of these structures shows an average number 1.88 per nucleus in Cap-H2 overexpressing cells and 0.04 per nucleus in cells not overexpressing Cap-H2 (C). This is a significant increase in frequency with Cap-H2 overexpression, *P*-value 7.1 e^−7^. Maximum diameter of these structures is not statistically different in Cap-H2 induction compared to those formed in the GAL4 control (D), *P*-value 0.59. Scale bar is 10 microns for all panels.

Increased condensin II activity leads to defects in nuclear architecture *in vivo* and in cultured *Drosophila* cells (Buster *et al.* 2013; Hartl *et al.* 2008). In an effort to systematically characterize these changes in the nuclear envelope, salivary glands from the heat shock−inducible Cap-H2 overexpression line (HS83-GFP-LacI, LacO(60F);Hsp70-GAL4, EY09979), referred to as HS > GAL4 UAS > Cap-H2, was compared with the GAL4 control line (HS83-GFP-LacI, LacO(60F);Hsp70-GAL4), HS > GAL4. These two lines are genetically identical except that the HS > GAL4 UAS > Cap-H2 line is capable of overexpressing Cap-H2 whereas HS > GAL4 is not (Hartl *et al.* 2008). In the HS > GAL4 UAS > Cap-H2 line, nuclear envelope shape is normal in the absence of heat shock induction (Figure 1A). In contrast, staining of the nuclear envelope with anti-Lamin revealed complex three-dimensional structures within the nucleus after Cap-H2 expression that we refer to as NR invaginations (Figure 1B and Figure S1). NR structures were continuous with or immediately adjacent to the nuclear envelope (Figure S1). An identical heat shock treatment of the HS > GAL4 line (not capable of overexpressing Cap-H2) rarely resulted in nuclear envelope defects (Figure 1, C and D and Figure 2C). Immunostaining with an antibody to the nuclear pore complex revealed that NR invaginations contained integral membrane proteins and stain positive for the membrane marker WGA (Figure 2). Strikingly, NR structures have a notable absence of DNA as well as histones and nuclear GFP (Figure 1B, Figure 2A, and Figure S2C). Similar results were obtained when an alternative Cap-H2 overexpression transgene, Cap-H2-eGFP, was driven by a tissue-specific 43B > GAL4 driver that is not heat shock dependent, indicating that neither heat stress nor the specific overexpression construct are contributing factors in the formation of these nuclear structures (Figure S3C). Taken together, these data fit the description of the NR, where the contents of the intranuclear vesicles are contiguous with the cytoplasm and/or excludes contents normally only found inside the nucleus (Malhas *et al.* 2011).

Nuclei from the HS > GAL4 UAS > Cap-H2 line without heat shock displayed no observable nuclear architecture defects (Figure 1A). Quantification of intra-nuclear structures after heat shock treatment in nuclei labeled with anti-Lamin showed a statistically significant increase in the presence of these structures with Cap-H2 overexpression as compared with the GAL4 control line (Figure 2C). The number of events per nucleus increased from 0.04 in the HS > GAL4 line, to 1.88 in the HS > GAL4 UAS > Cap-H2 line, yielding a p-value of 7.1 e^−7^. We emphasize that, although rare, these intranuclear structures are observed in wild-type nuclei that are not overexpressing Cap-H2 (Figure 2, C and D). Although greatly increased in frequency, the intranuclear structures observed in salivary glands in no other way appear to be different from those observed in control cells with normal levels of condensin activity, for instance, diameters of the structures were not different between treatments (Figure 2D).

Two different antibodies were used to visualize the nuclear membrane and NR, anti-Nup, and anti-Lamin. To confirm that NR detection does not vary depending on the method used, a direct comparison between the antibody stains was performed. The nuclear envelope of salivary glands was labeled with WGA and either anti-Nup or anti-Lamin in CapH 2 overexpression (Figure S4, A and B), and control GAL4 line (Figure S4, C and D). Quantification revealed no significant difference between the number of NR events detected with anti-Nup compared with anti-Lamin in the CapH 2 overexpression line (2.03 and 2.1 NR events per nucleus), nor in the GAL4 control (0.23 and 0.23 NR events per nucleus) (Figure S4E).

Examination of other tissues suggests that the formation of NR structures is not restricted to salivary gland nuclei. Heat shock induction of Cap-H2 in the HS > GAL4 UAS > Cap-H2 line induces similar structures in the diploid nuclei of the body wall muscles of third instar larvae (Figure S5, A and B). Nuclear envelope defects are also observed when Cap-H2 is overexpressed using a larval muscle specific driver (Figure S5, C and D).

**Figure 3:**
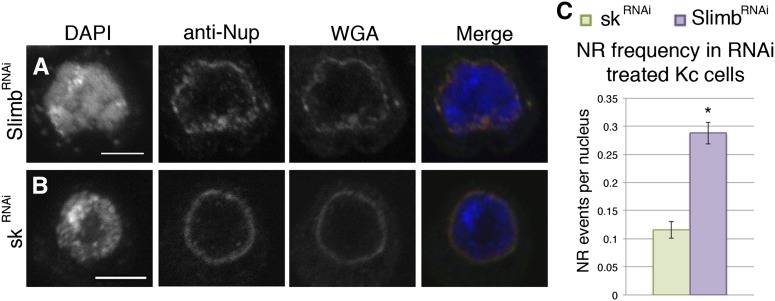
Cap-H2 stabilization leads to NR formation in cell culture. The nuclear membrane of Kc cell nuclei is visualized with wheat germ agglutinin (WGA) and antibody to the nuclear pore complex (anti-Nup). An increase in NR formation can be observed in cells with Cap-H2 stabilization, achieved through Slimb[RNAi] (A). Nuclei from control RNAi treatment maintain typical spherical configuration (B). Quantification of these structures shows significant increase in frequency with Cap-H2 stabilization (C), *P*-value 0.0025. Scale bars: 10 microns.

NR formation also was explored in a nonpolyploid model, specifically cells in culture. One effective method of increasing overall CapH 2 protein levels in cell culture is to reduce levels of Slimb, the ubiquitin ligase that targets CapH 2 for degradation. By reducing Slimb levels through RNAi, CapH 2 is stabilized and drives condensin II-mediated chromatin compaction (Buster *et al.* 2013). We used this method to evaluate the ability of CapH 2 to drive NR formation in cells. NR events and the nuclear membrane were detected using WGA and anti-Nup staining in control sk[RNAi]-treated cells, indicating that envelope remodeling is a normal occurrence in these cells (Figure 3, A and B). However, in cells treated with Slimb[RNAi], where CapH 2 protein is stabilized, NR events were increased by more than twofold (Figure 3, A and B). Notably, cells displayed a less-severe response to CapH 2 activity than salivary gland nuclei; however, this is not entirely unexpected, as the surface area, lipid content, and filament strength of a nucleus will vary between tissues; these factors will likely modulate the intrinsic properties of a cell’s nuclear envelope. Additionally, the two experiments relied on different methods for hyper-activation of CapH 2, one through overexpression, and the other through a stabilization of endogenous protein. These factors may lead to different amounts of CapH 2 activity and therefore result in varied severity of the phenotype. Nonetheless, a significant increase of NR formation was detected between sk[RNAi] and Slimb[RNAi] treatments, 0.12 compared with 0.29 NR events per nucleus, respectively (Figure 3C), suggesting that nuclear envelope remodeling through chromatin compaction is not restricted to highly polyploidy tissues.

### NR events are type II invaginations

**Figure 4:**
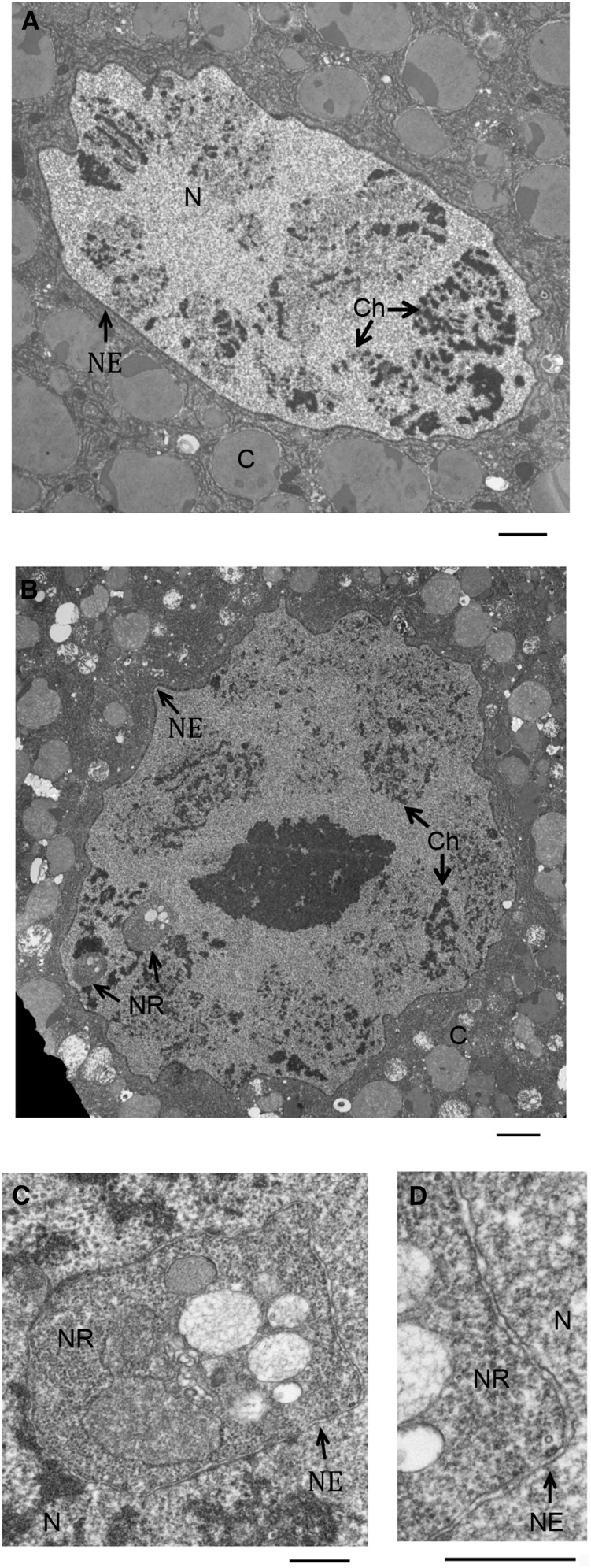
Transmission electron microscopy (TEM) imaging of nuclear envelope and nucleoplasmic reticulum (NR) in salivary gland nuclei. TEM of salivary glands reveal the ultrastructure of the nuclear envelope. Labeled in the panels are the following features: N (nucleus), C (cytoplasm), Ch (chromatin), NE (nuclear envelope), and NR (nucleoplasmic reticulum). The nuclear envelope can be seen lining the nuclear periphery of the GAL4 control (A). Cap-H2 overexpressing nuclei have additional structures within the nucleus, termed NR (B). Greater resolution images of the Cap-H2 overexpression nucleus, (C) and (D), reveal that the NR has an intact double membrane layer. Scale bars: (A) 2 microns, (B) 2 microns, (C) 500 nm, (D) 500 nm.

The structures of the nuclear envelope that constitute the NR have been separated into two classes; type I, and type II invaginations (Malhas *et al.* 2011). Type I invaginations contain only the nuclear lamina and inner membrane layer, requiring a delamination of the envelope’s two membranes. Alternatively, type II invaginations consist of the complete double membrane layer. Our observation that structures induced by condensin activation contain nuclear pore proteins as well as label positive for the WGA lectin suggested that they are double membrane structures (Figure 1 and Figure 2). To further validate whether these structures are NR invaginations and to identify the class of NR present in the salivary gland, we examined NR membrane structure. Transmission electron microscopy of a sectioned salivary gland was performed to view detailed lipid structures of the nuclear envelope. Control nuclei contained densely stained chromatin with characteristic polytene chromosome banding (Figure 4A). In contrast, NR structures were readily identified in Cap-H2 overexpressing salivary gland nuclei (Figure 4B). Close examination of the NR membrane revealed two distinct lipid bilayers, indicating that the double membrane layer was intact (Figure 4, C and D). Furthermore, densely-stained chromatin was never observed inside NR structures. We conclude that these structures conform to the previously described definition of NR structures, specifically type-II NR events (Malhas *et al.* 2011), and are consistent with DNA, histone and nuclear-GFP signals that are excluded from the interior of these membrane containing structures (Figure 1, Figure 2, and Figure S2).

### NR invaginations associate with chromatin-envelope tethers

**Figure 5:**
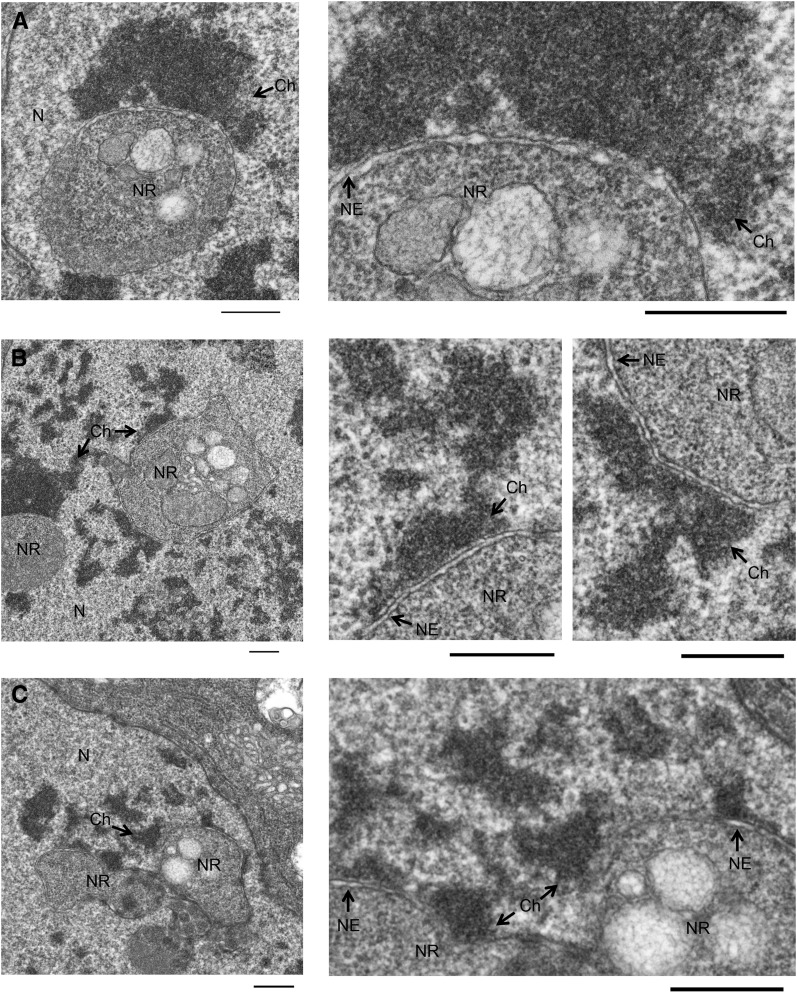
Chromatin associates with nucleoplasmic reticulum (NR) membrane. Transmission electron microscopy (TEM) of salivary glands reveal the ultrastructure of the nucleus in Cap-H2 overexpression tissues. Labeled in the panels are the following features: N (nucleus), C (cytoplasm), Ch (chromatin), NE (nuclear envelope), and NR (nucleoplasmic reticulum). Individual NR events are shown (A−C), with increased magnification on the right hand panels. In each instance, chromatin can be observed abutting the membrane of the NR. Scale bars are 500 nm in all panels.

**Figure 6:**
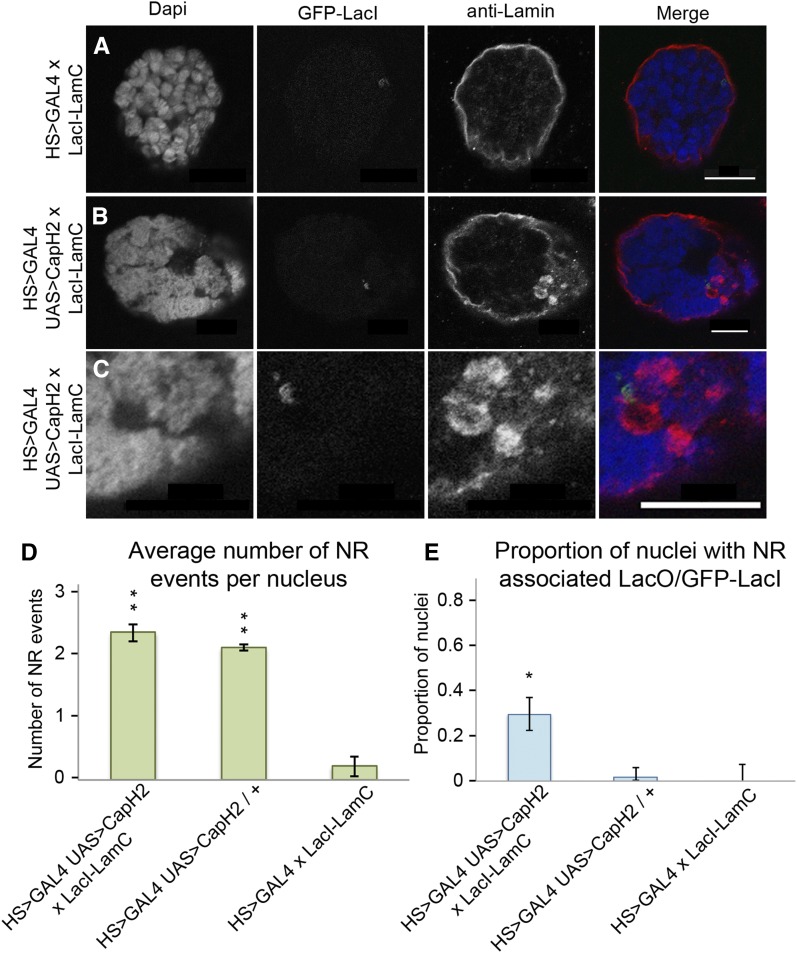
Chromatin-envelope tethers associate with nucleoplasmic reticulum (NR). Salivary gland nuclei were examined for effects of ectopically expressed chromatin-envelope tether in Cap-H2 overexpression and control. The nuclear envelope is visualized with antibody to the nuclear pore complex (anti-Nup) and the chromatin-envelope tether tracked with green fluorescent protein (GFP)-LacI. In the heat shock control nucleus (A), the LacO array can be seen localizing to the nuclear periphery. The Cap-H2 overexpression nucleus has alternate localization, with the LacO array in the nuclear interior and associating with NR event (B). NR event can be observed associating with LacO array in greater detail in a greater magnification view (C). Total number of NR events was quantified for each cross (D). Both Cap-H2 overexpression crosses had increased NR events compared to the GAL4 control line, p-value <2.2e^−16^. Yet, there was no statistical difference between the Cap-H2 overexpression nuclei with and without chromatin-envelope tether, *P*-value 0.252. The frequency of association of the LacO array to NR was elevated in the Cap-H2 overexpression with chromatin-envelope tether (E), *P*-value 0.0031. Scale bars are 10 microns in all panels.

Our model predicts that physical interactions or linkages between the chromatin and nuclear envelope are responsible for the alterations in the nuclear envelope following chromatin remodeling. Indeed, with very few exceptions, all type-II NR structures visualized by transmission electron microscopy associated with chromatin on the nuclear-proximal membrane layer (Figure 5). We emphasize that these type-II NR structures have clearly visible double membranes, consistent with the hypothesis that chromatin tethers in the nuclear envelope allow NR structures to be pulled into the nuclear interior. To test whether such attachments mediate NR events, we introduced an ectopic chromatin-lamin tether, as previously described (Dialynas *et al.* 2010). A LacI-LaminC fusion protein was expressed in a genetic background where a LacO array and GFP-LacI also were present, thus introducing a chromatin tether into our condensin overexpression system. If such a linkage facilitates the formation of NR invaginations, then we predict that the LacI-LaminC tethered DNA would associate with NR at greater frequency than the same region of DNA in the absence of the tether. Three genotypes were assayed for association of the LacO array with NR events: Cap-H2 overexpression (LacO, GFP-LacI; HS > GAL4, UAS > Cap-H2) crossed to flies with the tether construct, Cap-H2 overexpression (LacO, GFP-LacI; HS > GAL4, UAS > Cap-H2) crossed to flies without the tether construct, and GAL4 control (LacO, GFP-LacI; HS > GAL4) crossed to flies with the tether construct. In all genotypes, the same LacO arrays were present and visualized by a GFP-LacI fusion protein. As the GFP-LacI fusion protein is used for detection in place of LacI-antibody staining, only concentrated GFP-LacI was detectible, and therefore presented as a single spot at the LacO array (Buster *et al.* 2013; Hartl *et al.* 2008). Coexpression of GFP-LacI with LacI-LaminC did not interfere with formation of the tether, since the LacO array localized to the nuclear envelope. Frequency of NR events and the proportion of nuclei with the LacO array associating with an NR invagination were measured for each genotype (Figure 6). A statistically significant increase in LacO association with NR was observed for the HS > GAL4 UAS > Cap-H2 line with tether, occurring in 33% of nuclei compared with 1.6% of nuclei in HS > GAL4 UAS > Cap-H2 without tether and 0% of nuclei in HS > GAL4 with tether. Although the frequency of LacO-associated NR events was dramatically greater in the Cap-H2 overexpression line with a LacI-LaminC tether, there was no difference in the frequency of total NR events between lines with or without the LacI-LaminC tether (Figure 6). This argues that the LacI-LaminC itself does not affect the frequency of NR events, and strongly suggests that LacI-LaminC tether-associated NR events were not random. Thus, we conclude that condensin II compaction forces act on chromatin-envelope tethers to form NR structures.

### NR originates from the nuclear envelope

**Figure 7:**
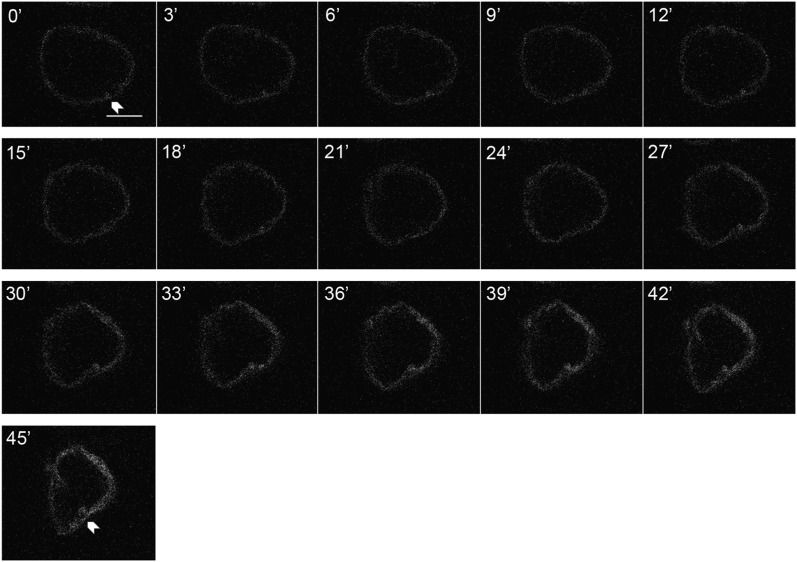
Time-lapse imaging of nucleoplasmic reticulum (NR) formation in Cap-H2 overexpressing salivary gland nucleus. Time lapse imaging of the nuclear envelope in Cap-H2 overexpressing nucleus used a fluorescent nuclear envelope, marked with a green fluorescent protein (GFP) tagged nuclear pore complex. Images are displayed in three-minute increments. The nucleus was imaged by capturing z-optical slices, with a step size of 2 microns, images shown are of a single z-slice. Structural changes of the nuclear envelope can be observed throughout the course of the experiment, including the budding of NR from the nuclear envelope at t = 45’. Arrowhead indicates the location of the NR budding event at initial and final time points. Scale bar is 10 microns. See File S1.

The mechanism of NR formation is currently unknown. We postulate that the NR is a remodeling of the nuclear envelope and therefore originates from the nuclear envelope. However, it is possible that these structures assemble *de novo* and are assembled inside the nucleus. To directly address this question, we used GFP-tagged nuclear pore protein Nup107 to observe NR events in live salivary gland cells. After heat shock induction of Cap-H2, glands were time lapse imaged for 45 min. Z-slices were taken to capture three-dimentional structures of the nucleus. During the imaging session, changes in nuclear shape accompanied thickening of the nuclear membrane and eventual internalized budding of the envelope (Figure 7, Figure S6, and File S1). Control time lapses failed to reveal the formation of similar structures (Figure S7 and File S2). Interestingly, some NR invaginations already had formed when imaging was initiated and then resorbed back into the envelope, supporting the idea that NR events are reversible and dynamic (Figure S8 and File S3). It is important to note that because structures were imaged with z-slices, and that the nucleus remains dynamic during imaging, some features move between individual z-slices.

### Perturbing the structural integrity of the nuclear envelope enhances NR events

**Figure 8:**
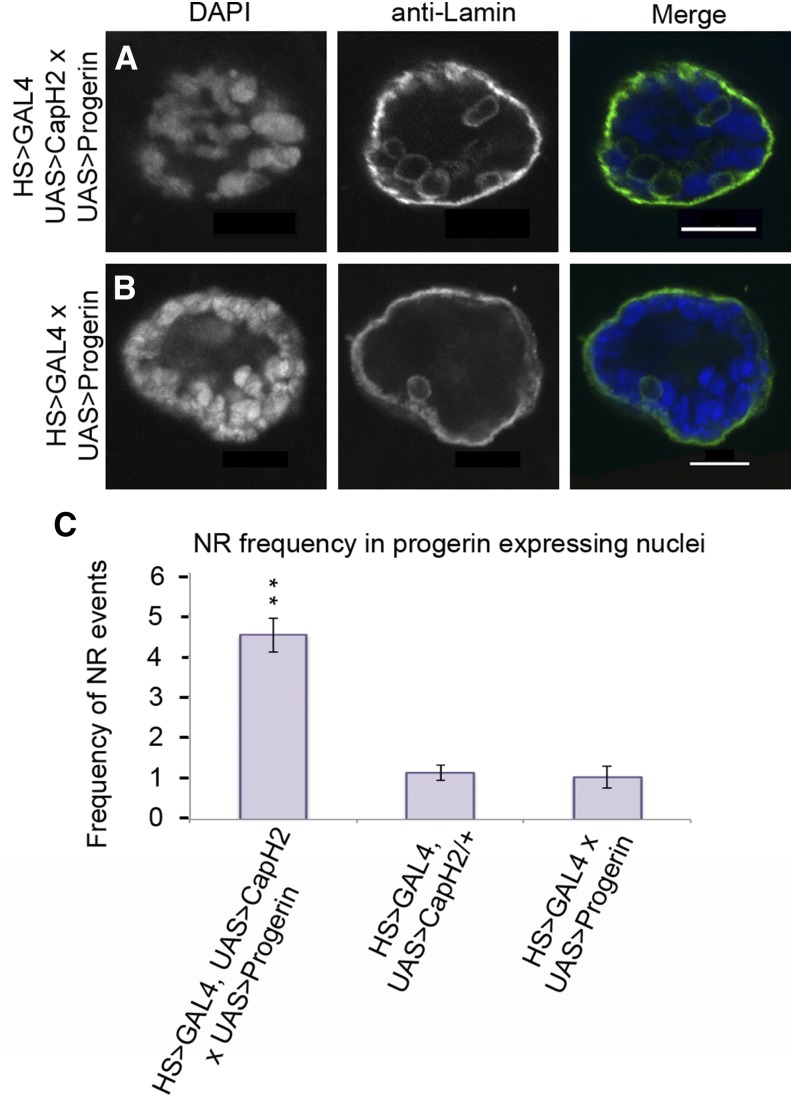
Weakening of the nuclear envelope enhances Cap-H2 induction of nucleoplasmic reticulum. Nuclear defects of individual salivary glands overexpressing the human progerin protein are visualized with DAPI and anti-Lamin. Overexpression of Cap-H2 yields an enhanced nucleoplasmic reticulum (NR) phenotype (A), compared to GAL4 control (B). Quantification of NR events in progerin background can increase the phenotype when paired with Cap-H2 overexpression (C). A significant increase is observed for overexpression of Cap-H2 and progerin compared to Cap-H2 overexpression alone, *P*-value 4.15e^−7^. Scale bars are 10 microns for all panels.

**Figure 9:**
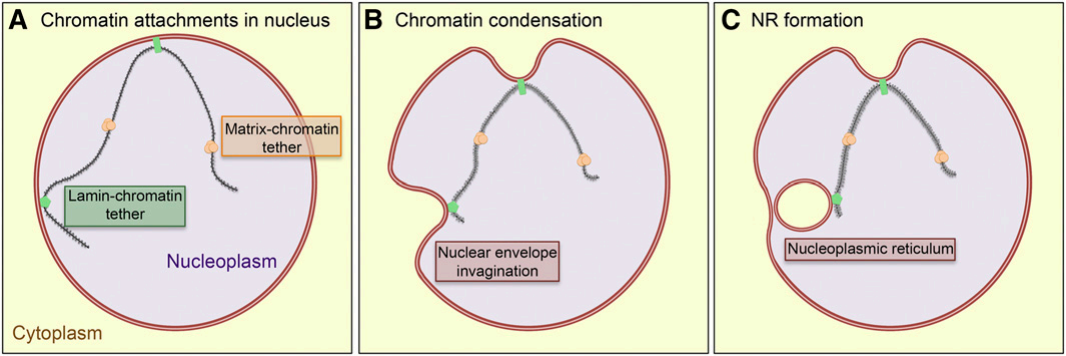
Model of chromatin compaction leading to nucleoplasmic reticulum (NR) formation. (A) Attachments between the chromatin (black and gray line) and nuclear envelope (red line) are established and maintained for normal cellular function. (B) As chromatin compaction shortens the chromatin, the nuclear envelope is pulled into the nuclear space through these attachments. (C) Formation of the NR occurs as the result of one or more chromatin-envelope attachments.

Previous studies have suggested that nuclear shape and size are maintained by a coordinated balance of forces derived from chromatin condensation inside the nucleus and cytoskeletal dynamics outside the nucleus, both converging on the nuclear envelope (Buster *et al.* 2013; Maniotis *et al.* 1997; Rowat *et al.* 2008). The mechanical stress of these forces is distributed through the nuclear envelope, with the nuclear lamina adding further structural stability and mediating the interaction of these forces. We postulated that if mechanical forces provided by chromatin compaction contributes to NR formation, then disrupting the integrity of the nuclear envelope would likely increase the NR phenotype, as the structural integrity of the lamin filaments would be less able to counterbalance these internal mechanical forces. To test this, we expressed the human disease causing lamin protein, progerin by itself and in combination with Cap-H2 overexpression. Control cells expressing progerin alone exhibited 1.0 NR events per nucleus (Figure 8). Likewise, Cap-H2 overexpression alone exhibited 1.14 NR events per nucleus (Figure 8). However, coexpression of both proteins increased the frequency to 4.49 NR events per nucleus (Figure 8). Based on the results, we have generated a model illustrating how these various forces may be acting to remodel the nuclear architecture (Figure 9).

## Discussion

Nuclear architecture has significant influence over the function of the nucleus. Beyond its role as an organelle boundary, the nuclear envelope plays a dynamic role in gene regulation and genomic organization. The localization of a gene to the nuclear periphery can have profound impacts on its transcriptional state or mRNA export efficiency. Therefore, the complex network of nuclear envelope that constitutes the NR may have functional significance, as it brings chromatin in the nuclear interior closer in proximity to the inner nuclear envelope. Moreover, defects and instability of the nuclear architecture have been directly linked to multiple human diseases, and aberrant regulation of the NR has been observed in several disease models (Malhas *et al.* 2011). Thus, understanding the dynamic regulation of the NR is relevant to human disease.

It has been proposed that forces shape the nuclear envelope and changes in these forces can lead to nuclear defects. These forces, originating from the cytoskeleton and balancing properties of the lamina matrix, collectively contribute to erecting the higher order structure of the nuclear envelope. Here, we propose that an additional force acts upon the nuclear envelope: the mechanical stress exerted from chromosome condensation and genomic reorganization in interphase cells.

Our findings indicate that NR formation in *Drosophila* can occur in unperturbed cells, albeit rarely, and can be mediated by condensin II activity, with significant increase in NR events following induction of Cap-H2. Additionally, we have shown that condensin II-mediated NR events are type II invaginations, indicating that the double membrane layer of the nuclear envelope is reshaped to protrude into the nucleoplasm. Live cell analysis, combined with the temporal control of our heat shock expression lines, revealed that the NR is a distortion of the nuclear envelope. These results suggest that NR formation is a dynamic process and that the nuclear envelope is plastic, as it is not restricted to the shape it takes on when the envelope reforms. Previous studies have been unable to temporally resolve NR events. Although it would be difficult to generalize these findings to all NR formation, it is nonetheless pertinent that nuclear architecture can change in response to chromatin state and, in particular, that it is responsive to mechanical forces emanating from within the nucleus.

Given the many ways in which chromatin and the nuclear envelope physically interact, we postulated that physical linkages may be responsible for the previously observed NR structures. Experiments that tethered chromatin to the nuclear envelope predictably localized DNA to the nuclear periphery. However, upon heat shock induction of Cap-H2, NR formation was frequently associated with tether attachments. These findings implicate similar endogenous chromatin-envelope interactions in NR formation and are consistent with our electron micrographs that reveal endogenous chromatin-NR contacts. We speculate that these contacts are focal points of NR biogenesis. A recent study has computationally predicted that *Drosophila* polytene chromosomes have approximately 48 chromatin-envelope contacts, many more than previously thought (Kinney *et al.* 2014). This suggests therefore that many regions of the genome could function as tethers that drive NR biogenesis.

Finally, structural weakening of the envelope is able to enhance the NR phenotype because progerin expression increases NR events. Our findings support a model whereby nuclear architecture is maintained by a delicate balancing of counteracting forces. By disrupting the nuclear lamina, the nuclear envelope becomes more susceptible to mechanical forces, such as those derived from chromatin reorganization. Specifically, we propose that chromatin-envelope tethers transduce pulling forces from chromatin condensation that reposition envelope into the nuclear interior and restructure nuclear architecture. This study and work from others have begun to elucidate the complexity of factors acting on the nuclear envelope (Dahl *et al.* 2005; Goulbourne *et al.* 2011; Mazumder and Shivashankar 2007; Mazumder *et al.* 2008). The cumulative data suggest a highly dynamic nuclear envelope, responding to biological processes throughout interphase. Continuing work on the biophysics of the NR as well as the genetic factors that regulate these structures will yield valuable insight into the maintenance of the nuclear envelope as well as its function and role in normal and disease states.

## Supplementary Material

Supporting Information
