# Supplementary material for: Condensins Exert Force on Chromatin-Nuclear Envelope Tethers to Mediate Nucleoplasmic Reticulum Formation in Drosophila melanogaster
Source: G3 (Bethesda). 2014 Dec 30;5(3):341–52. doi: 10.1534/g3.114.015685 (PMC4349088; doi:10.1534/g3.114.015685)
Supplement: Supporting Information [file supp_5_3_341__index.html]

Condensins Exert Force on Chromatin-Nuclear Envelope Tethers to Mediate Nucleoplasmic Reticulum Formation in Drosophila melanogaster — Supporting Information 

# Condensins Exert Force on Chromatin-Nuclear Envelope Tethers to Mediate Nucleoplasmic Reticulum Formation in *Drosophila melanogaster*

## Supporting Information for Bozler *et al.*, 2015

**Files in this Data Supplement:**

- Supporting Information - Figures S1-S8, Table S1, and Files S1-S5 (PDF, 689 KB)
- Figure S1 - Three-dimensional imaging of nucleoplasmic reticulum. (PDF, 337 KB)
- Figure S2 - Nucleoplasmic reticulum excludes nuclear contents. (PDF, 171 KB)
- Figure S3 - Tissue specific expression of Cap-H2 induces NR formation. (PDF, 130 KB)
- Figure S4 - Comparison of NR detection methods. (PDF, 181 KB)
- Figure S5 - Nuclear architecture changes in muscle nuclei induced by Cap-H2 overexpression. (PDF, 147 KB)
- Figure S6 - Z-stacks of initial time point for live imaging of nucleoplasmic reticulum formation. (PDF, 279 KB)
- Figure S7 - Time lapse imaging of control salivary gland nucleus. (PDF, 147 KB)
- Figure S8 - Time lapse imaging of pre-formed nucleoplasmic reticulum. (PDF, 158 KB)
- Table S1 - Fly stocks used in experiments. (PDF, 137 KB)
- File S4 - Supplemental Materials and Methods (PDF, 111 KB)
- File S1 - Supplementary video 1 (.m4v, 739 KB)
- File S2 - Supplementary video 2 (.m4v, 1 MB)
- File S3 - Supplementary video 3 (.mp4, 1 MB)
- File S5 - Raw data. (.xlsx, 44 KB)
