# Supplementary material for: Condensins Exert Force on Chromatin-Nuclear Envelope Tethers to Mediate Nucleoplasmic Reticulum Formation in Drosophila melanogaster
Source: G3 (Bethesda). 2014 Dec 30;5(3):341–52. doi: 10.1534/g3.114.015685 (PMC4349088; doi:10.1534/g3.114.015685)
Supplement: Supporting Information [file supp_g3.114.015685_015685SI.pdf]

G3 · Genes | Genomes | Genetics

Condensins exert force on chromatin-nuclear envelope tethers to mediate nucleoplasmic reticulum formation in *Drosophila melanogaster*

Julianna Bozler[1], Huy Q Nguyen[1], Gregory C Rogers[2] and Giovanni Bosco[1]

[1] Geisel School of Medicine at Dartmouth, Hanover, New Hampshire, United States of America
[2] Department of Cellular and Molecular Medicine, University of Arizona Cancer Center, University of Arizona, Tucson, Arizona, United States of America

Corresponding author:
Giovanni Bosco
Giovanni.Bosco@Dartmouth.Edu
Geisel School of Medicine at Dartmouth
Department of Genetics
7400 Remsen Hanover, NH 03755

DOI: 10.1534/g3.114.015685

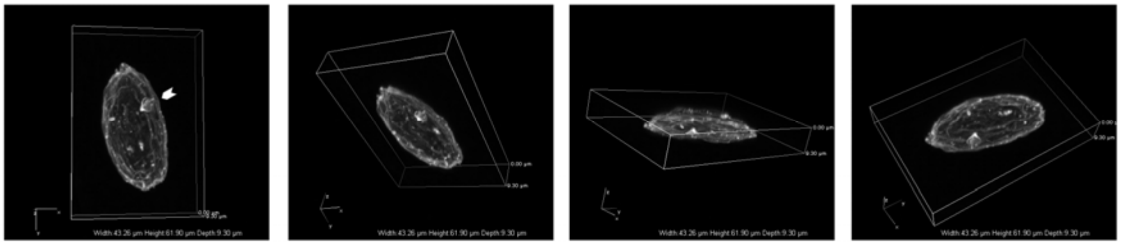

Figure S1 Three-dimensional imaging of nucleoplasmic reticulum. A three-dimensional projection of a Cap-H2 overexpressing nucleus was created from confocal z-slices, step size 0.5 microns. The nuclear envelope was labeled with anti-Lamin. The NR, marked by arrowhead, can be seen projecting into the interior of the nucleus. The three-dimensional rendition is rotated for various viewing angles.

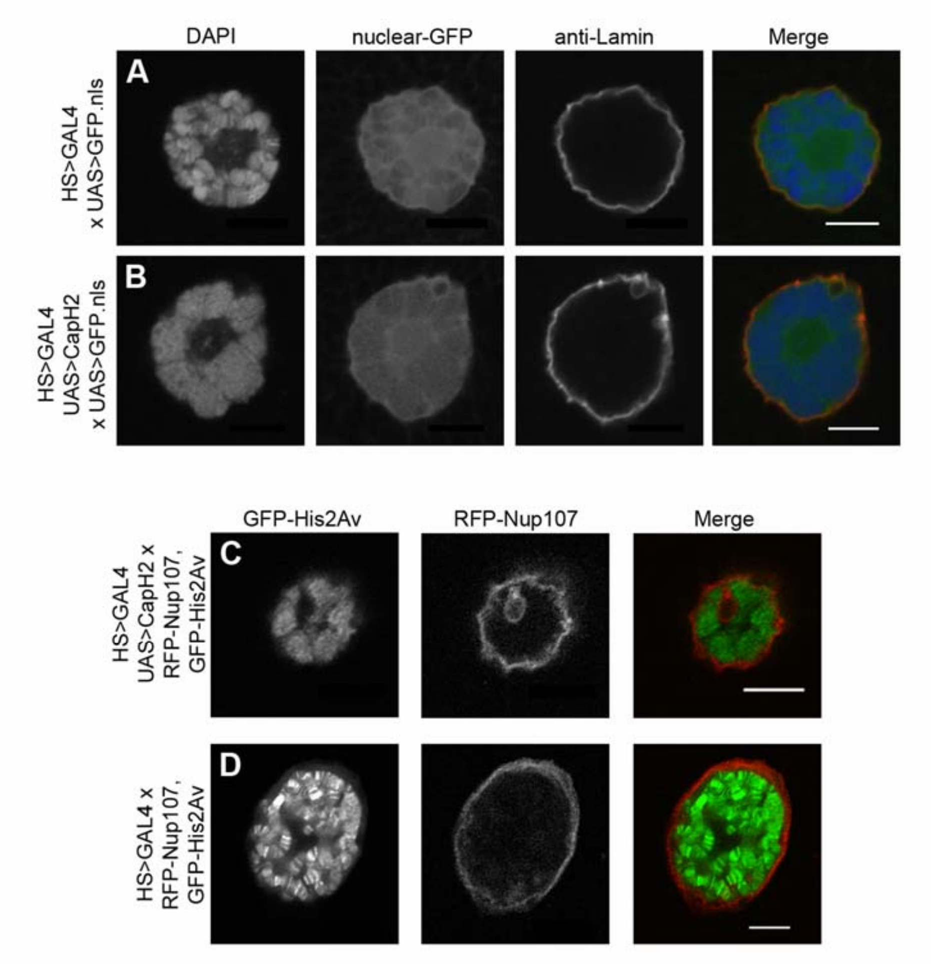

Figure S2 Nucleoplasmic reticulum excludes nuclear contents. Individual salivary gland nuclei are imaged in Cap-H2 overexpression and control lines. Boundaries of the nuclear envelope are marked either with anti-Lamin (A-B), or by a RFP tagged nuclear pore complex (C-D). Nuclear localizing GFP can be seen diffuse throughout the nucleus in GAL4 control (A). GFP signal is diffuse through Cap-H2 overexpression nucleus, but excluded from interior of the nucleoplasmic reticulum (B). Similarly, GFP-histone is excluded from the nucleoplasmic reticulum (C), and appears chromatin bound in Cap-H2 overexpression (C) and GAL4 control (D). Scale bars are 10 microns in all panels.

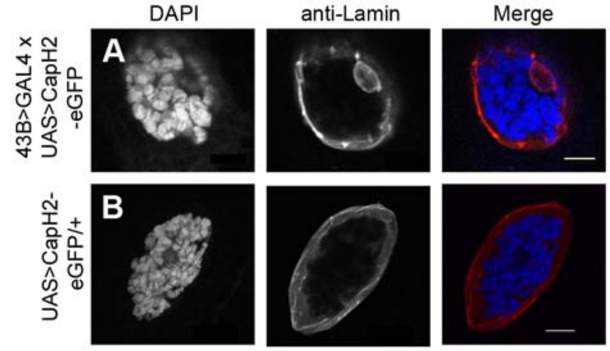

Figure S3 Tissue specific expression of Cap-H2 induces NR formation. The tissue specific driver 43B was used to drive overexpression of Cap-H2, without the need for heat shock. The nuclear envelope is marked by anti-Lamin. NR formation can be seen in Cap-H2 overexpression, with nuclear envelope structures protruding into the nuclear space (A). GAL4 control nucleus shows typical lamin staining of the nuclear envelope (B). Scale bars are 10 microns in both panels.

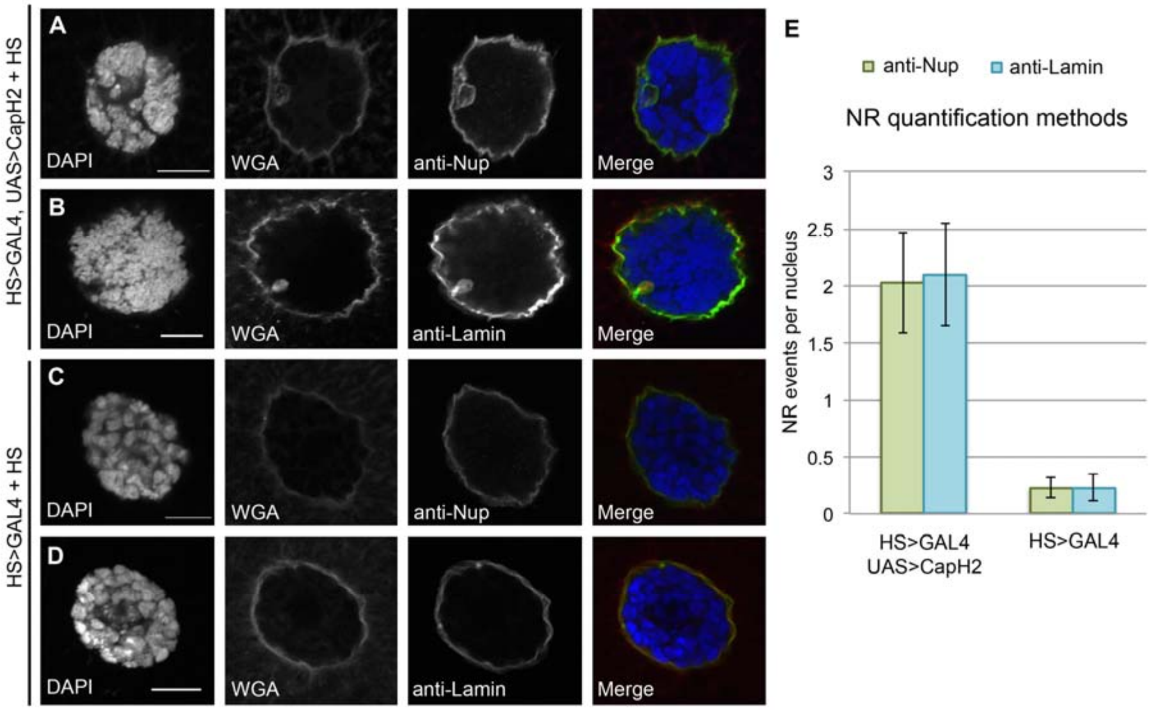

Figure S4 Comparison of NR detection methods. Nuclear membrane and NR is detected in CapH2 overexpressing salivary gland nuclei, induced by heat shock, using antibody to the nuclear pore complex (anti-Nup) (A), and antibody to lamin (anti-Lamin) (B). The nuclear membrane in the GAL4 control line was also detected with anti-Nup (C), and anti-Lamin (D). The nuclear envelope is counter stained with wheat germ agglutinin (WGA), and DNA detected with DAPI. Quantification of NR reveals no significant difference between antibody detection methods (E), p-values: CapH2 overexpression (0.92), GAL4 control (1). Scale bar is10 microns for all panels.

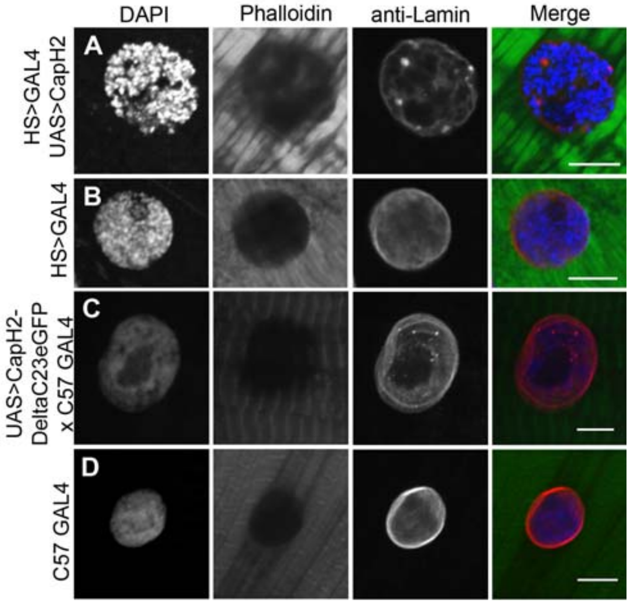

Figure S5 Nuclear architecture changes in muscle nuclei induced by Cap-H2 overexpression. Individual muscle nuclei from the larval body wall were imaged with endogenous Cap-H2 overexpression (A) and overexpression of a stabilized Cap-H2 protein (C), along with the corresponding GAL4 control (B, D). Anti-Lamin was used to mark the nuclear envelope. Spherical protrusions of the nuclear envelope into the nuclear space can be seen in Cap-H2 overexpression through heat shock induction (A). Smaller perturbations of the envelope are seen with tissue specific expression of Cap-H2 (C). Scale bars are 10 microns in all panels.

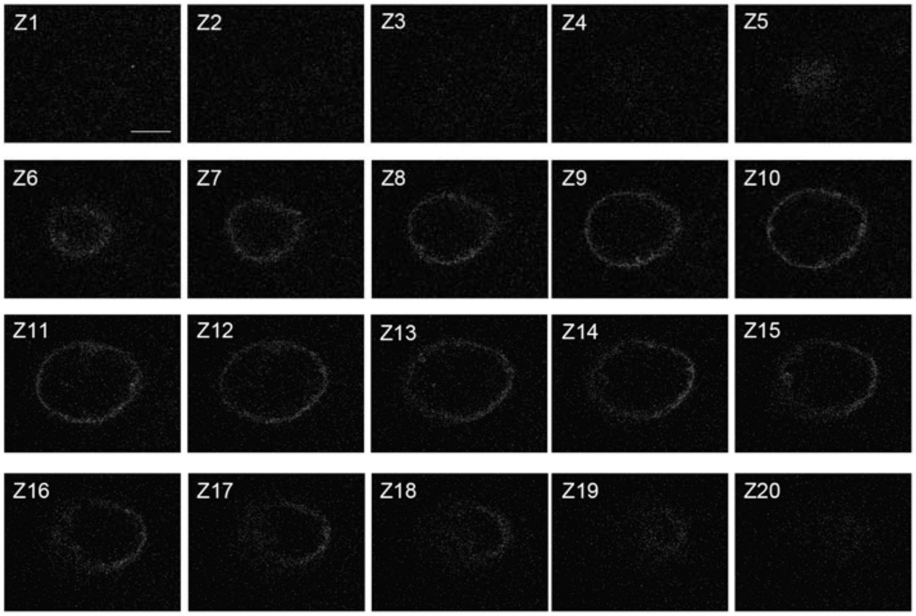

Figure S6 Z-stacks of initial time point for live imaging of nucleoplasmic reticulum formation. Live imaging of the nuclear envelope in Cap-H2 overexpressing nucleus utilized a fluorescent nuclear envelope, marked with a GFP tagged nuclear pore complex. Images are z-slices with step size of 2 microns from initial time point of time lapse imaging of NR formation. Scale bar is 10 microns.

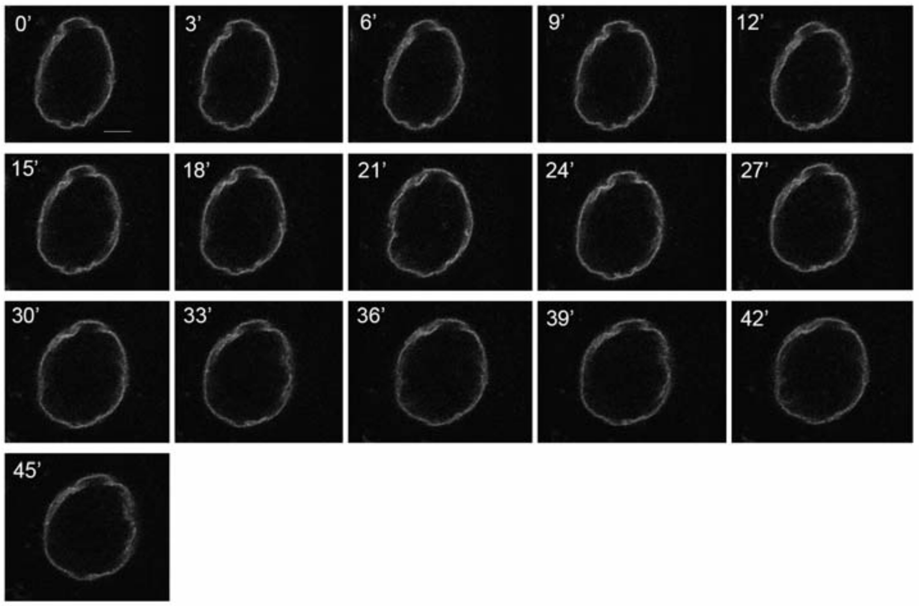

Figure S7 Time lapse imaging of control salivary gland nucleus. Time lapse imaging of the nuclear envelope without heat shock induction of Cap-H2. Imaging relied on a fluorescent nuclear envelope, marked with a GFP tagged nuclear pore complex. Images are displayed in three-minute increments. Minimal structural changes are observed over the course of the experiment. Scale bar is 10 microns. See supplementary video 2.

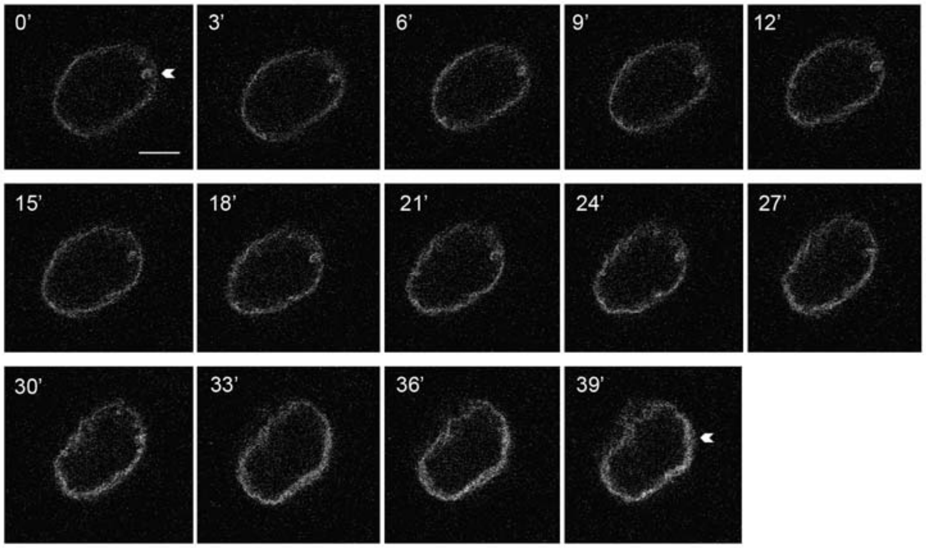

Figure S8 Time lapse imaging of pre-formed nucleoplasmic reticulum. Time lapse imaging of the nuclear envelope with Cap-H2 induction utilized a fluorescent nuclear envelope, marked with a GFP tagged nuclear pore complex. Images are displayed in three-minute increments. At the start of imaging, NR was present, with its location indicated by arrowhead at t=0. Through the time lapse, the NR appears to fuse with the nuclear envelope and disappears. The NR starting location is indicated at t=45 by arrowhead. Scale bar is 10 microns. See supplementary video 3.

Table S1 Fly stocks used in experiments.

Abbreviated name	Genotype	Source
HS>GAL4, UAS>CapH2	W*;HS83-GFP-LacI, LacO (60F);Hsp70-GAL4, EY09979	
HS>GAL4	W*;HS83-GFP-LacI, LacO (60F);Hsp70-GAL4	
CapH2-eGFP	UAS-CapH2-eGFP.1	
CapH2-deltaC23-eGFP	UAS-CapH2-ΔC23-eGFP.3	
C57 GAL4	P{GawB}C57	Wallrath Lab
GFP-Nup107	W*; P{w[+mc]=GFP-Nup107.k}9.1	Bloomington; #35514
GFP-Nup107; HS>GAL4 UAS>CapH2	W*; P{w[+mc]=GFP-Nup107.k}9.1; EY09979, Hsp70-GAL4	
UAS-progerin	W*;Δ150$127;Tm6B/MR15	Tree Lab
43B-GAL4	W*;P{GawB}43B	
LacI-LamC	W[-], hs-act-LacO-hsp26/plant-hsp70/white-4D5; LacI-Lamin C	Wallrath Lab
UAS-GFP.nls	W[1118]; P{w[+mc]=UAS-GFP.nls}8	Bloomington; #4776
RPG-Nup107, H2Av-GFP	W*;Nup107[E8]/CyO; P{w[+mC]=mRFP-Nup107.K}7.1, P{w[+mC]=His2Av[T:Avic\GFP-S65T]}62A	Bloomington; #35518

Files S1-S3

Available for download as .mp4 files at http://www.g3journal.org/lookup/suppl/doi:10.1534/g3.114.015685/-/DC1

File S1 Supplementary video 1

File S2 Supplementary video 2

File S3 Supplementary video 3

File S4

Supplemental Materials and Methods

TEM Protocol

Samples were fixed with 2% gluteraldehyde and 2% paraformaldehyde in 0.1 NaCacodylate (460 mOsm). Salivary glands were fixed at room temperature for 2 hours, and transferred to 4°C for overnight fixation. Fix was removed and samples washed three times with 0.1M NaCacodylate and 0.15M Sucrose (350 mOsm). Postfix incubation was carried out with 2% osmium tetraoxide in 0.1M NaCacodylate and 0.07M Sucrose (350 mOsm) for one hour. Samples were rinsed with 0.1M NaCacodylate in 0.15 M Sucrose (350 mOsm), and washed with distilled water three times. En-Bloc stain was carried out in 2% uranyl acetate aqueous solution in the dark for one hour. Following distilled water washes, samples were dehydrated through an ethanol series of 30%, 50%, 70%, 85% and 95% for 15 minutes each. Three one-hour incubations in 100% ethanol completed dehydration step. Samples were incubated in propylene oxide for two 30-minute periods, and then transferred to a 1.5:1 solution of LX112:PO for six hours. Samples were then placed in vacuum desiccantor overnight. Initial polymerization occurred at 45 ^{o}C overnight, and then transferred to 60 ^{o}C for an additional 24 hours. Blocks were allowed to cool and sectioned. Ultrathin sections were stained with uranyl acetate aqueous solution for 20 minutes, and lead citrate for 5 minutes.

File S5

Raw Data

Available for download as an Excel file at http://www.g3journal.org/lookup/suppl/doi:10.1534/g3.114.015685/-/DC1
