# Supplementary material for: Condensins Exert Force on Chromatin-Nuclear Envelope Tethers to Mediate Nucleoplasmic Reticulum Formation in Drosophila melanogaster
Source: G3 (Bethesda). 2014 Dec 30;5(3):341–52. doi: 10.1534/g3.114.015685 (PMC4349088; doi:10.1534/g3.114.015685)
Supplement: Supporting Information [file supp_g3.114.015685_FigureS1.pdf]

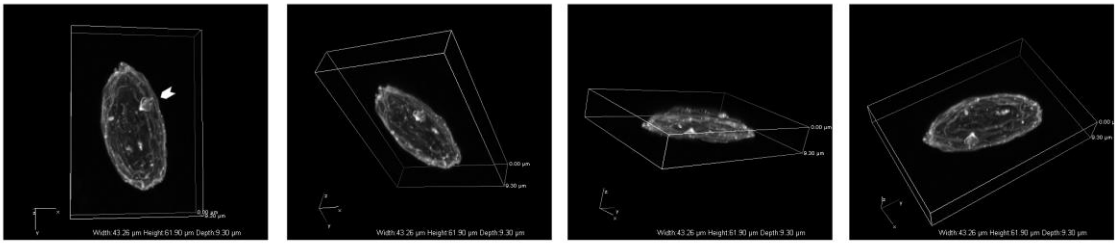

Figure S1 Three-dimensional imaging of nucleoplasmic reticulum. A three-dimensional projection of a Cap-H2 overexpressing nucleus was created from confocal z-slices, step size 0.5 microns. The nuclear envelope was labeled with anti-Lamin. The NR, marked by arrowhead, can be seen projecting into the interior of the nucleus. The three-dimensional rendition is rotated for various viewing angles.
