# Supplementary material for: Condensins Exert Force on Chromatin-Nuclear Envelope Tethers to Mediate Nucleoplasmic Reticulum Formation in Drosophila melanogaster
Source: G3 (Bethesda). 2014 Dec 30;5(3):341–52. doi: 10.1534/g3.114.015685 (PMC4349088; doi:10.1534/g3.114.015685)
Supplement: Supporting Information [file supp_g3.114.015685_FigureS2.pdf]

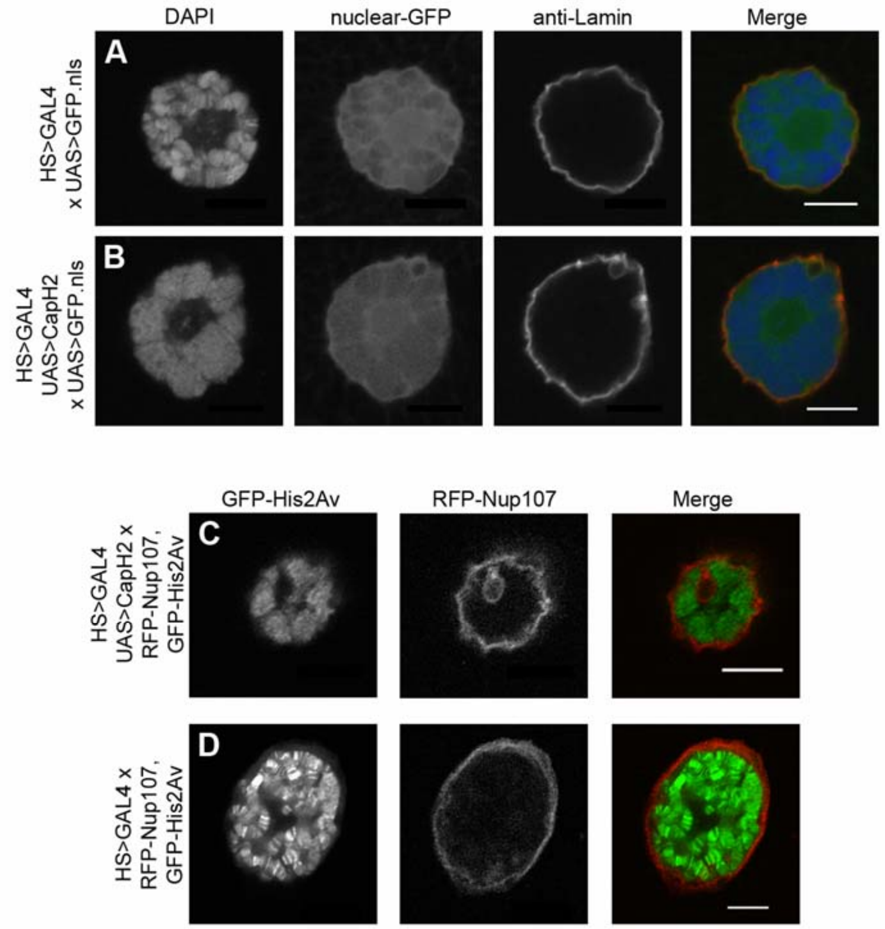

Figure S2 Nucleoplasmic reticulum excludes nuclear contents. Individual salivary gland nuclei are imaged in Cap-H2 overexpression and control lines. Boundaries of the nuclear envelope are marked either with anti-Lamin (A-B), or by a RFP tagged nuclear pore complex (C-D). Nuclear localizing GFP can be seen diffuse throughout the nucleus in GAL4 control (A). GFP signal is diffuse through Cap-H2 overexpression nucleus, but excluded from interior of the nucleoplasmic reticulum (B). Similarly, GFP-histone is excluded from the nucleoplasmic reticulum (C), and appears chromatin bound in Cap-H2 overexpression (C) and GAL4 control (D). Scale bars are 10 microns in all panels.
