# Supplementary material for: Condensins Exert Force on Chromatin-Nuclear Envelope Tethers to Mediate Nucleoplasmic Reticulum Formation in Drosophila melanogaster
Source: G3 (Bethesda). 2014 Dec 30;5(3):341–52. doi: 10.1534/g3.114.015685 (PMC4349088; doi:10.1534/g3.114.015685)
Supplement: Supporting Information [file supp_g3.114.015685_FigureS3.pdf]

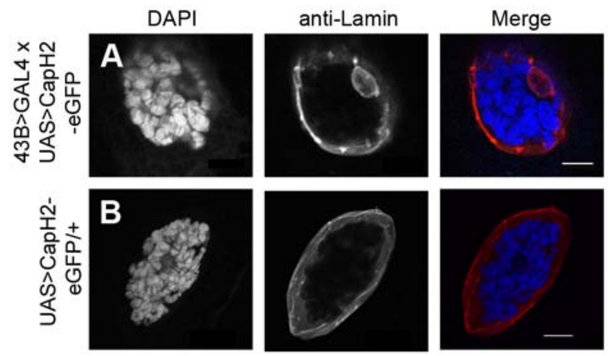

Figure S3 Tissue specific expression of Cap-H2 induces NR formation. The tissue specific driver 43B was used to drive overexpression of Cap-H2, without the need for heat shock. The nuclear envelope is marked by anti-Lamin. NR formation can be seen in Cap-H2 overexpression, with nuclear envelope structures protruding into the nuclear space (A). GAL4 control nucleus shows typical lamin staining of the nuclear envelope (B). Scale bars are 10 microns in both panels.
