# Supplementary material for: Condensins Exert Force on Chromatin-Nuclear Envelope Tethers to Mediate Nucleoplasmic Reticulum Formation in Drosophila melanogaster
Source: G3 (Bethesda). 2014 Dec 30;5(3):341–52. doi: 10.1534/g3.114.015685 (PMC4349088; doi:10.1534/g3.114.015685)
Supplement: Supporting Information [file supp_g3.114.015685_FigureS4.pdf]

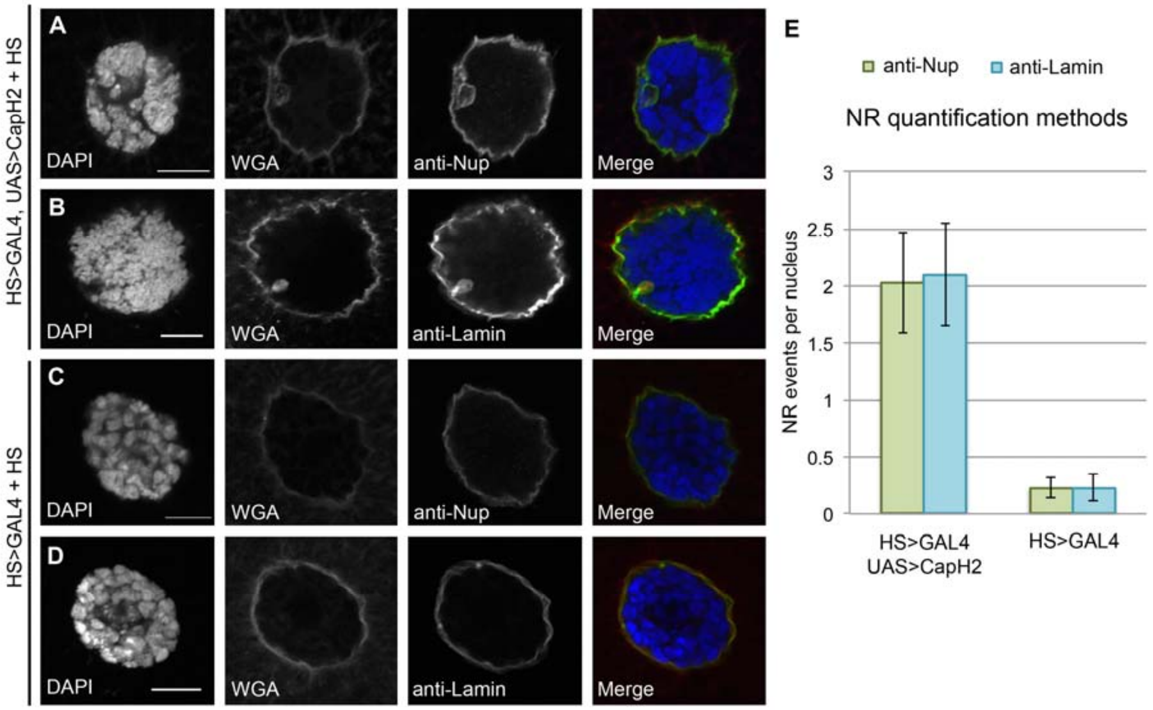

Figure S4 Comparison of NR detection methods. Nuclear membrane and NR is detected in CapH2 overexpressing salivary gland nuclei, induced by heat shock, using antibody to the nuclear pore complex (anti-Nup) (A), and antibody to lamin (anti-Lamin) (B). The nuclear membrane in the GAL4 control line was also detected with anti-Nup (C), and anti-Lamin (D). The nuclear envelope is counter stained with wheat germ agglutinin (WGA), and DNA detected with DAPI. Quantification of NR reveals no significant difference between antibody detection methods (E), p-values: CapH2 overexpression (0.92), GAL4 control (1). Scale bar is10 microns for all panels.
