# Supplementary material for: Condensins Exert Force on Chromatin-Nuclear Envelope Tethers to Mediate Nucleoplasmic Reticulum Formation in Drosophila melanogaster
Source: G3 (Bethesda). 2014 Dec 30;5(3):341–52. doi: 10.1534/g3.114.015685 (PMC4349088; doi:10.1534/g3.114.015685)
Supplement: Supporting Information [file supp_g3.114.015685_FigureS5.pdf]

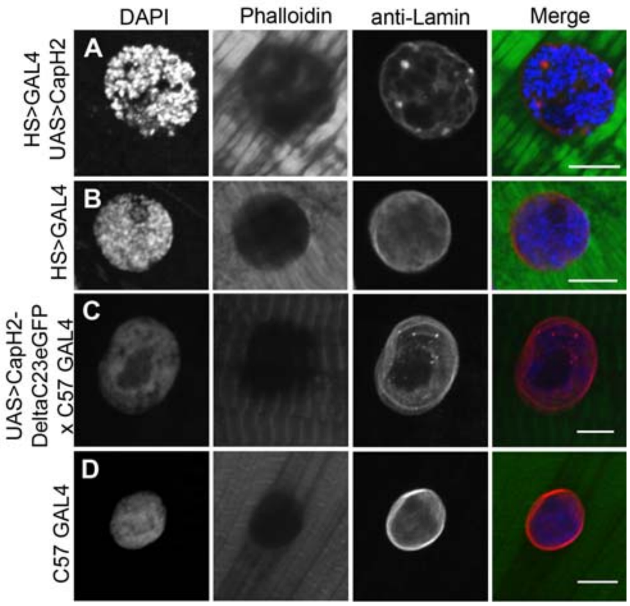

Figure S5 Nuclear architecture changes in muscle nuclei induced by Cap-H2 overexpression. Individual muscle nuclei from the larval body wall were imaged with endogenous Cap-H2 overexpression (A) and overexpression of a stabilized Cap-H2 protein (C), along with the corresponding GAL4 control (B, D). Anti-Lamin was used to mark the nuclear envelope. Spherical protrusions of the nuclear envelope into the nuclear space can be seen in Cap-H2 overexpression through heat shock induction (A). Smaller perturbations of the envelope are seen with tissue specific expression of Cap-H2 (C). Scale bars are 10 microns in all panels.
