# Supplementary material for: Condensins Exert Force on Chromatin-Nuclear Envelope Tethers to Mediate Nucleoplasmic Reticulum Formation in Drosophila melanogaster
Source: G3 (Bethesda). 2014 Dec 30;5(3):341–52. doi: 10.1534/g3.114.015685 (PMC4349088; doi:10.1534/g3.114.015685)
Supplement: Supporting Information [file supp_g3.114.015685_FigureS6.pdf]

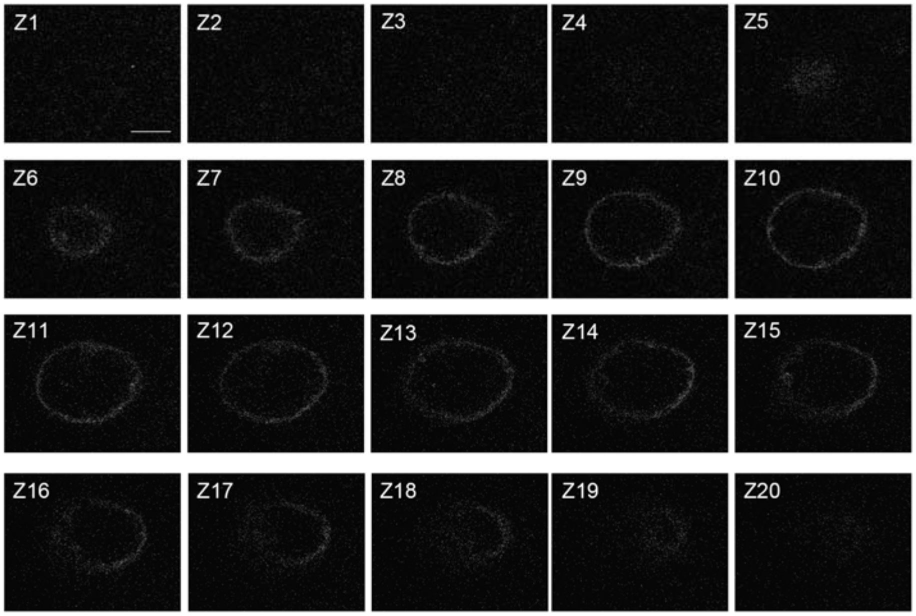

Figure S6 Z-stacks of initial time point for live imaging of nucleoplasmic reticulum formation. Live imaging of the nuclear envelope in Cap-H2 overexpressing nucleus utilized a fluorescent nuclear envelope, marked with a GFP tagged nuclear pore complex. Images are z-slices with step size of 2 microns from initial time point of time lapse imaging of NR formation. Scale bar is 10 microns.
