# Supplementary material for: Condensins Exert Force on Chromatin-Nuclear Envelope Tethers to Mediate Nucleoplasmic Reticulum Formation in Drosophila melanogaster
Source: G3 (Bethesda). 2014 Dec 30;5(3):341–52. doi: 10.1534/g3.114.015685 (PMC4349088; doi:10.1534/g3.114.015685)
Supplement: Supporting Information [file supp_g3.114.015685_FigureS7.pdf]

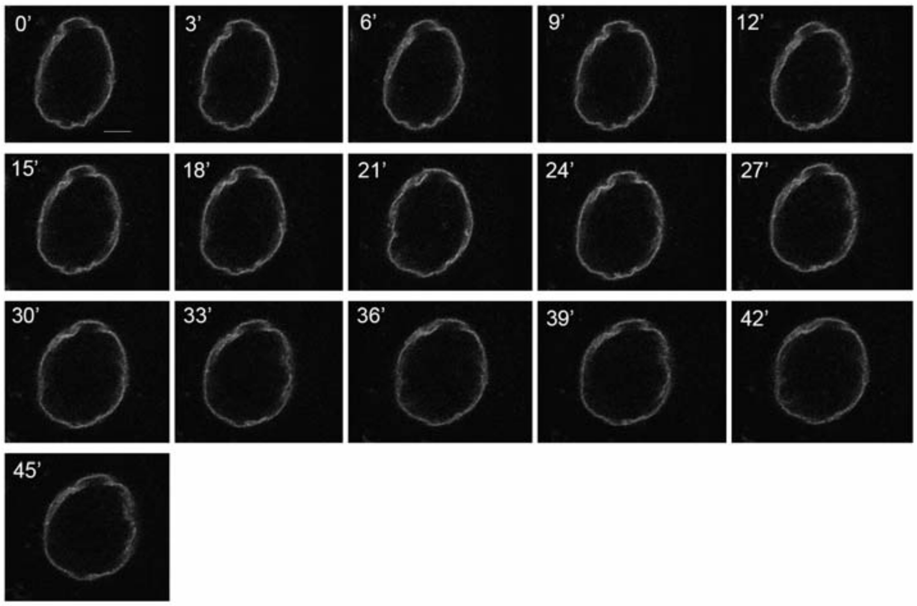

Figure S7 Time lapse imaging of control salivary gland nucleus. Time lapse imaging of the nuclear envelope without heat shock induction of Cap-H2. Imaging relied on a fluorescent nuclear envelope, marked with a GFP tagged nuclear pore complex. Images are displayed in three-minute increments. Minimal structural changes are observed over the course of the experiment. Scale bar is 10 microns. See supplementary video 2.
