# Supplementary material for: Condensins Exert Force on Chromatin-Nuclear Envelope Tethers to Mediate Nucleoplasmic Reticulum Formation in Drosophila melanogaster
Source: G3 (Bethesda). 2014 Dec 30;5(3):341–52. doi: 10.1534/g3.114.015685 (PMC4349088; doi:10.1534/g3.114.015685)
Supplement: Supporting Information [file supp_g3.114.015685_FigureS8.pdf]

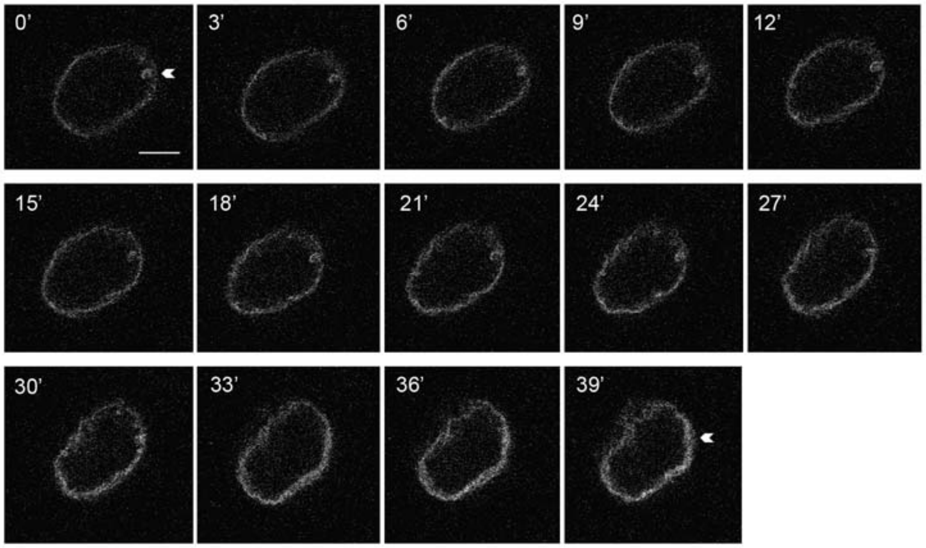

Figure S8 Time lapse imaging of pre-formed nucleoplasmic reticulum. Time lapse imaging of the nuclear envelope with Cap-H2 induction utilized a fluorescent nuclear envelope, marked with a GFP tagged nuclear pore complex. Images are displayed in three-minute increments. At the start of imaging, NR was present, with its location indicated by arrowhead at t=0. Through the time lapse, the NR appears to fuse with the nuclear envelope and disappears. The NR starting location is indicated at t=45 by arrowhead. Scale bar is 10 microns. See supplementary video 3.
