# Supplementary material for: Condensins Exert Force on Chromatin-Nuclear Envelope Tethers to Mediate Nucleoplasmic Reticulum Formation in Drosophila melanogaster
Source: G3 (Bethesda). 2014 Dec 30;5(3):341–52. doi: 10.1534/g3.114.015685 (PMC4349088; doi:10.1534/g3.114.015685)
Supplement: Supporting Information [file supp_g3.114.015685_TableS1.pdf]

Table S1 Fly stocks used in experiments.

Abbreviated name	Genotype	Source
HS>GAL4, UAS>CapH2	W*;HS83-GFP-LacI, LacO (60F);Hsp70-GAL4, EY09979	
HS>GAL4	W*;HS83-GFP-LacI, LacO (60F);Hsp70-GAL4	
CapH2-eGFP	UAS-CapH2-eGFP.1	
CapH2-deltaC23-eGFP	UAS-CapH2-ΔC23-eGFP.3	
C57 GAL4	P{GawB}C57	Wallrath Lab
GFP-Nup107	W*; P{w[+mc]=GFP-Nup107.k}9.1	Bloomington; #35514
GFP-Nup107; HS>GAL4 UAS>CapH2	W*; P{w[+mc]=GFP-Nup107.k}9.1; EY09979, Hsp70-GAL4	
UAS-progerin	W*;Δ150$127;Tm6B/MR15	Tree Lab
43B-GAL4	W*;P{GawB}43B	
LacI-LamC	W[-], hs-act-LacO-hsp26/plant-hsp70/white-4D5; LacI-Lamin C	Wallrath Lab
UAS-GFP.nls	W[1118]; P{w[+mc]=UAS-GFP.nls}8	Bloomington; #4776
RPG-Nup107, H2Av-GFP	W*;Nup107[E8]/CyO; P{w[+mC]=mRFP-Nup107.K}7.1, P{w[+mC]=His2Av[T:Avic\GFP-S65T]}62A	Bloomington; #35518
