# Supplementary material for: Condensins Exert Force on Chromatin-Nuclear Envelope Tethers to Mediate Nucleoplasmic Reticulum Formation in Drosophila melanogaster
Source: G3 (Bethesda). 2014 Dec 30;5(3):341–52. doi: 10.1534/g3.114.015685 (PMC4349088; doi:10.1534/g3.114.015685)
Supplement: Supporting Information [file supp_g3.114.015685_FileS4.pdf]

File S4

Supplemental Materials and Methods

TEM Protocol

Samples were fixed with 2% gluteraldehyde and 2% paraformaldehyde in 0.1 NaCacodylate (460 mOsm). Salivary glands were fixed at room temperature for 2 hours, and transferred to 4°C for overnight fixation. Fix was removed and samples washed three times with 0.1M NaCacodylate and 0.15M Sucrose (350 mOsm). Postfix incubation was carried out with 2% osmium tetraoxide in 0.1M NaCacodylate and 0.07M Sucrose (350 mOsm) for one hour. Samples were rinsed with 0.1M NaCacodylate in 0.15 M Sucrose (350 mOsm), and washed with distilled water three times. En-Bloc stain was carried out in 2% uranyl acetate aqueous solution in the dark for one hour. Following distilled water washes, samples were dehydrated through an ethanol series of 30%, 50%, 70%, 85% and 95% for 15 minutes each. Three one-hour incubations in 100% ethanol completed dehydration step. Samples were incubated in propylene oxide for two 30-minute periods, and then transferred to a 1.5:1 solution of LX112:PO for six hours. Samples were then placed in vacuum desiccantor overnight. Initial polymerization occurred at 45 °C overnight, and then transferred to 60 °C for an additional 24 hours. Blocks were allowed to cool and sectioned. Ultrathin sections were stained with uranyl acetate aqueous solution for 20 minutes, and lead citrate for 5 minutes.
